# DFT-ML-Based Property Prediction of Transition Metal Complex Photosensitizers for Photodynamic Therapy

**DOI:** 10.1021/acsomega.5c08727

**Published:** 2025-10-31

**Authors:** Jingxing Gao, Yachao Dong, Tian Qiu, Wen Sun, Jian Du

**Affiliations:** School of Chemical Engineering, Dalian University of Technology, Dalian 116024, China

## Abstract

Photodynamic therapy (PDT) is a noninvasive clinical treatment for cancers using photosensitizers and light. While most research has focused on organic molecules, such as porphyrins as photosensitizers, there is emerging interest in the utilization of transition metal complexes (TMCs). Photosensitizer synthesis and the following performance test are time- and resource-consuming, so presynthetic screening of photosensitizers for their property would be critical. In this work, a hybrid mechanistic and data-driven model is proposed for the quantitative structure–property relationship (QSPR) of photosensitizers; important excited-state quantum chemistry descriptors (e.g., excitation energy) are first calculated based on density functional theory (DFT), and these descriptors, together with other molecular descriptors, are used to build single and hybrid machine learning (ML) models for the prediction of the singlet oxygen quantum yield of hexacoordinate TMC photosensitizers (Ru-, Ir-, and Re-complex). The support vector regression model and kernel ridge regression model are shown to provide good predictions on test (*R* ^2^ > 0.9) and external test sets (*R* ^2^ > 0.7) in single-ML models, while the delta-learning model and the Mixture-of-Experts model can further improve the generalization ability (*R* ^2^ up to 0.87 on the external test set) and show strong universality. SHAP analysis further confirms the reasonable choice of the mechanistic descriptors in the QSPR model. To our knowledge, this constitutes the first integrated DFT-ML framework specifically designed for the unique challenges of small data sets in TMC photosensitizer research.

## Graphical abstract

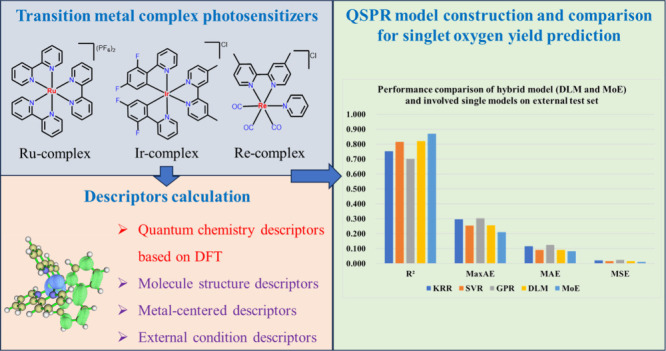

## 1 Introduction

Cancer is one of the major diseases of great threat to human health. The global cancer cases are expected to grow to 28.4 million cases in 2040, with a 47% increase over 2020.^1^ At present, the main therapies for cancer include surgical therapy,^2^ chemotherapy,^3^ radiotherapy,^4^ gene therapy,^5^ photodynamic therapy (PDT),^6^ and photothermal therapy (PTT).^7^ Among the above therapies, PDT is considered to be effective for superficial cancerous tissues because of its advantages of low toxicity, lack of drug resistance, and mild adverse reactions.^8^

The main process of PDT is to use light sources to activate nontoxic or microtoxic photosensitizers to produce cytotoxic reactive oxygen species (ROS), thereby inducing apoptosis and necrosis of cells at the tumor site. As shown in Figure 1, under the irradiation of appropriate wavelength light, the photosensitizer (PS) will be excited to a singlet state, and then quickly converted to a triplet state through intersystem crossing (ISC). Then, triplet state PS reacts with the substrate photodynamically to produce ROS. At present, this photodynamic process is divided into two types: type I and type II.^9–12^ In the process of type I photochemical reaction, PS in the triplet state reacts with nearby substrates to form radical cations or radical anions through electron transfer, which will further react with oxygen-containing substrates (such as water, oxygen, etc.) to produce ROS (such as superoxide anions and hydroxyl radicals).^13,14^ In type II photochemical reactions, PS in the triplet state directly transfers energy to oxygen to form highly reactive singlet oxygen (^1^O_2_), as shown in Figure 2.^15^ Therefore, PS is the core element of PDT, and its photophysical and chemical properties determine the therapeutic effect. There is now emerging interest in extending the use to transition metal complexes (TMC), which can display intense absorptions in the visible region, and many also possess high two-photon absorption cross sections, enabling two-photon excitation with NIR light. Therefore, transition metal complexes have become efficient candidates for PSs with developing potential.^16^

**Figure 1:**
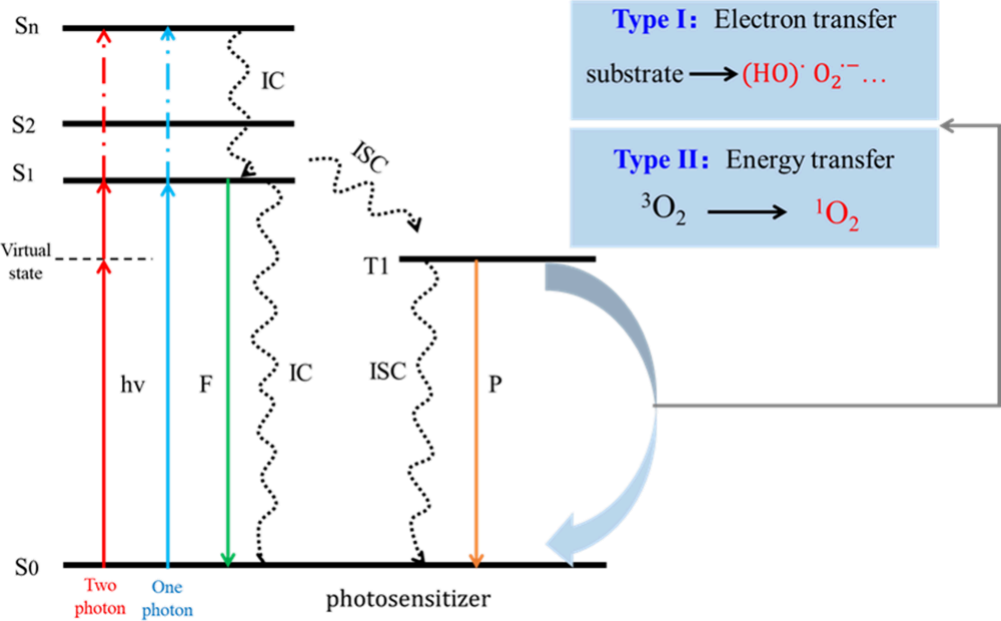
Jablonski energy level diagram for PDT.

**Figure 2:**
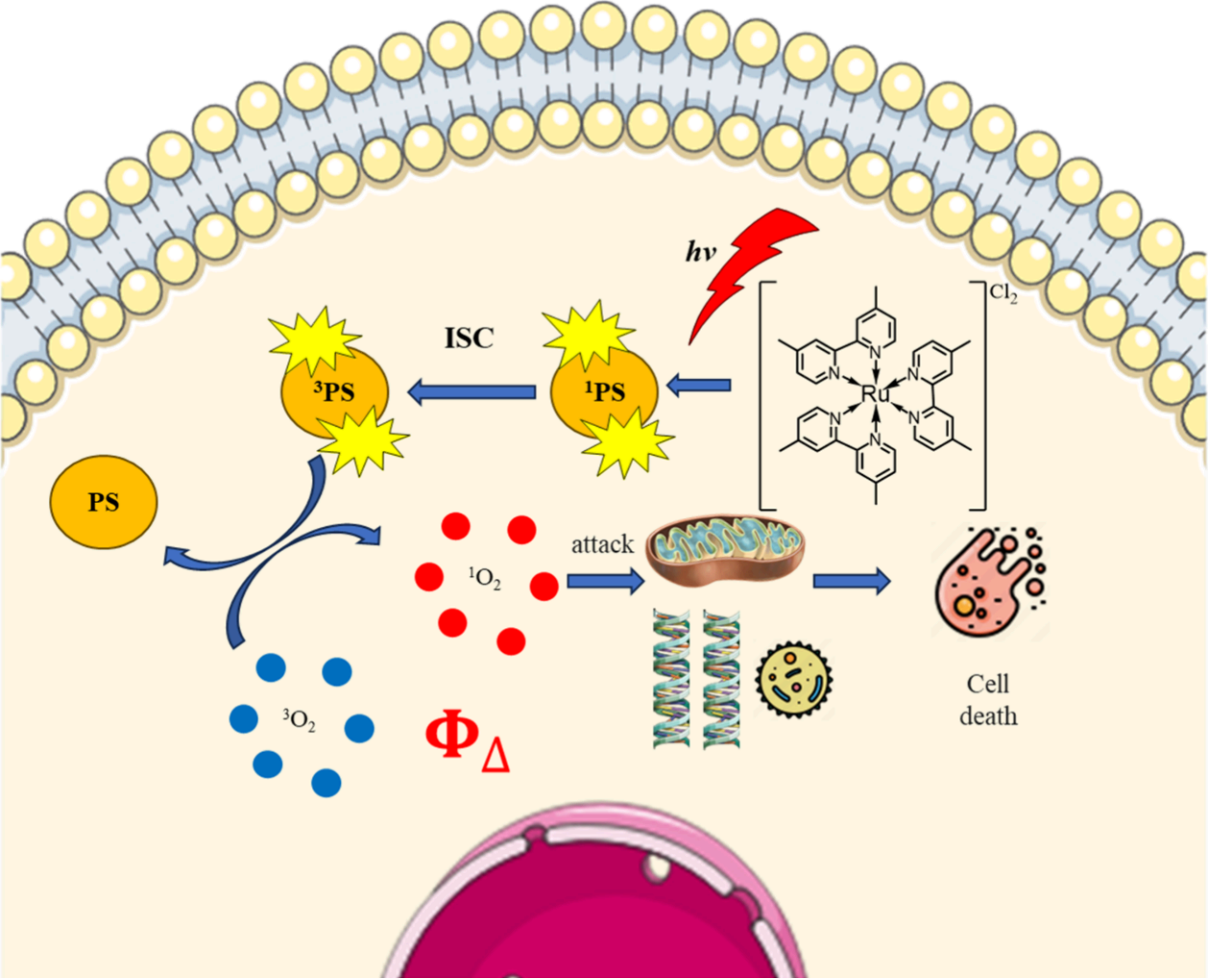
Schematic illustration of the process of the Ru complex in type II PDT.

The most studied transition metal in PDT has been Ru,^17,18^ which usually has high water solubility compared with porphyrins or phthalocyanines as well as a high Φ_Δ_, which is essential as a type II PDT PS.^19^ For example, a Ru (II) complex named “TLD1433” entered clinical trials in early 2017.^20^ A series of water-soluble Ru (II) phthalocyanines with large and stable conjugated π systems have been developed that enable efficient energy and electron transfer processes;^21^ a new generation series of cyclometalated Ru­(II) polypyridyl complexes have been designed and synthesized with the photophysical properties of revealed absorption maxima around 560 nm with an absorption up to 700 nm.^22^ Ir-complexes also have relatively wide applications in the field of photosensitizers. Organic-modified mesoporous silica nanoparticles containing iridium complexes have been synthesized, which exhibit photophysical properties such as high photoreaction yield and high singlet oxygen quantum yield.^23^ Two novel cyclometalated Ir-complexes have also been developed, which have strong emission peaks, long excited state lifetimes, and high singlet oxygen particle yields.^24^ There are currently very limited studies on rhenium complex photosensitizers, but they also have development potential; for example, a tricarbonyl Recomplex with endoplasmic reticulum-targeting activity has been designed and synthesized, which has strong absorption and a high singlet oxygen quantum yield.^25^

However, photosensitizer synthesis and the following determination of its photosensitizing properties are time- and resource-consuming processes. Therefore, preliminary and presynthetic screening of sensitizers for their ability to generate ^1^O_2_ would be of great value. Recently, mathematical modeling method^26,27^ and machine learning (ML) method^28–32^ have gained popularity and proved to be a powerful tool in various areas, which use algorithms to learn from data, detect patterns, and make fast and accurate predictions.^33–36^ ML has already been used in property prediction of organic molecule photosensitizers, including related properties with type I^37^ and type II PDT.^38,39^ A quantitative structure property relationship (QSPR) model has been established for a data set containing 32 porphyrins and metalloporphyrins.^40^ A new machine learning method has been developed to efficiently and accurately predict the emission energy and photoluminescent quantum yield.^41^ 15 single models and three different hybrid models have been proposed to evaluate a data set of 3,066 organic materials to predict photophysical properties (absorption wavelength, emission wavelength, and quantum yield).^42^ However, these models are not suitable for TMC photosensitizers because traditional structure descriptors such as SMILES can hardly capture all the information on such PSs, and the lack of data makes it difficult to use deep learning models such as a graph neural network. To the best of our knowledge, there is no research reporting the property prediction method of TMC photosensitizers for PDT. Thus, it is necessary to develop machine learning models with a small data set to predict the properties of TMC photosensitizers such as the triplet state lifetime τ_T_, the triplet quantum yield Φ_T*,*_ and the singlet oxygen quantum yield Φ_Δ_. DFT can elucidate intrinsic mechanisms that cannot be observed by experimental techniques and is widely used in theoretical chemistry.^43–45^ The combined DFT and ML method could be an excellent method for property prediction of TMC photosensitizers.

In this work, we introduce a systematic DFT-ML framework explicitly developed for the small-data regime prevalent in TMC photosensitizer development, which provides a tailored solution for accelerating the discovery of TMC photosensitizers. We study various single- and hybrid-ML models to predict the singlet oxygen quantum yield Φ_Δ_ of TMC, which is an evaluation index of type II photosensitizers for PDT; additionally, Φ_Δ_ is more important and easier to collect from the literature compared to the triplet state lifetime and the triplet quantum yield. In order to characterize the structure and charge transition under light irradiation of TMC photosensitizers during the PDT process, density functional theory (DFT) is chosen to calculate the properties of the excited state as the quantum chemistry descriptor, which could provide low-dimensional ML models suitable for the small data sets of TMC available in the literature. These models, based on quantum chemistry descriptors and other descriptors, are trained and tested on TMC data sets including Ru-complexes, Ir-complex, and Recomplex because they are the majority of reported TMCs, and they are all hexa-coordination TMCs and have similar structural characteristics. The result is compared with the performance of two hybrid-ML models, including the delta-learning model (DLM) and Mixture-of-Experts model (MoE), trained on a specific TMC photosensitizer data set to test whether the generalized metal model trained on three six-coordination TMC can replace the specialized metal model trained on TMC of a given metal center. The subsequent SHAP analysis shows descriptor contribution to the predicted Φ_Δ,_ which could provide strong interpretability of the proposed model, such as the excitation energy of the S1 state and T1 state. Based on the modeling results, the proposed DFT-MoE model has been found to be most accurate, which could provide theoretical support for experimental synthesis and screening.

The article is structured as follows. In Section 2, we introduce the calculation method of four types of descriptors and the details of six single-ML models and two hybrid-ML models. In Section 3, we analyze and compare the training results of these models and test the performance of the best models on a separate Ru-complex data set and Ir-complex data set. In Section 4, we make the concluding remarks.

## 2 Method

In this section, we first present the TMC photosensitizers used in the data set of the machine learning model training process; then, we introduce the calculation method of descriptors; finally, we give the details of the single and hybrid-ML models proposed in this work.

### 2.1 Data Set Construction and Preprocessing

Our data set consists of 136 sets of TMC photosensitizers data from different references. We collect the structures, solvents, and irradiation wavelengths in Φ_Δ_ test and corresponding Φ_Δ_ from these references (the data with Φ_Δ_ less than 0.01 and those with excessive differences in Φ_Δ_ only under different wavelength irradiations were removed in the preprocessing process). The properties distribution of the data set is shown in Figure 3. Additionally, the external test set is made up of the other 11 sets of data from references to test the generalization ability of the proposed models. The detailed information on the data set and the external test set is shown in Tables S1 and S2.

**Figure 3:**
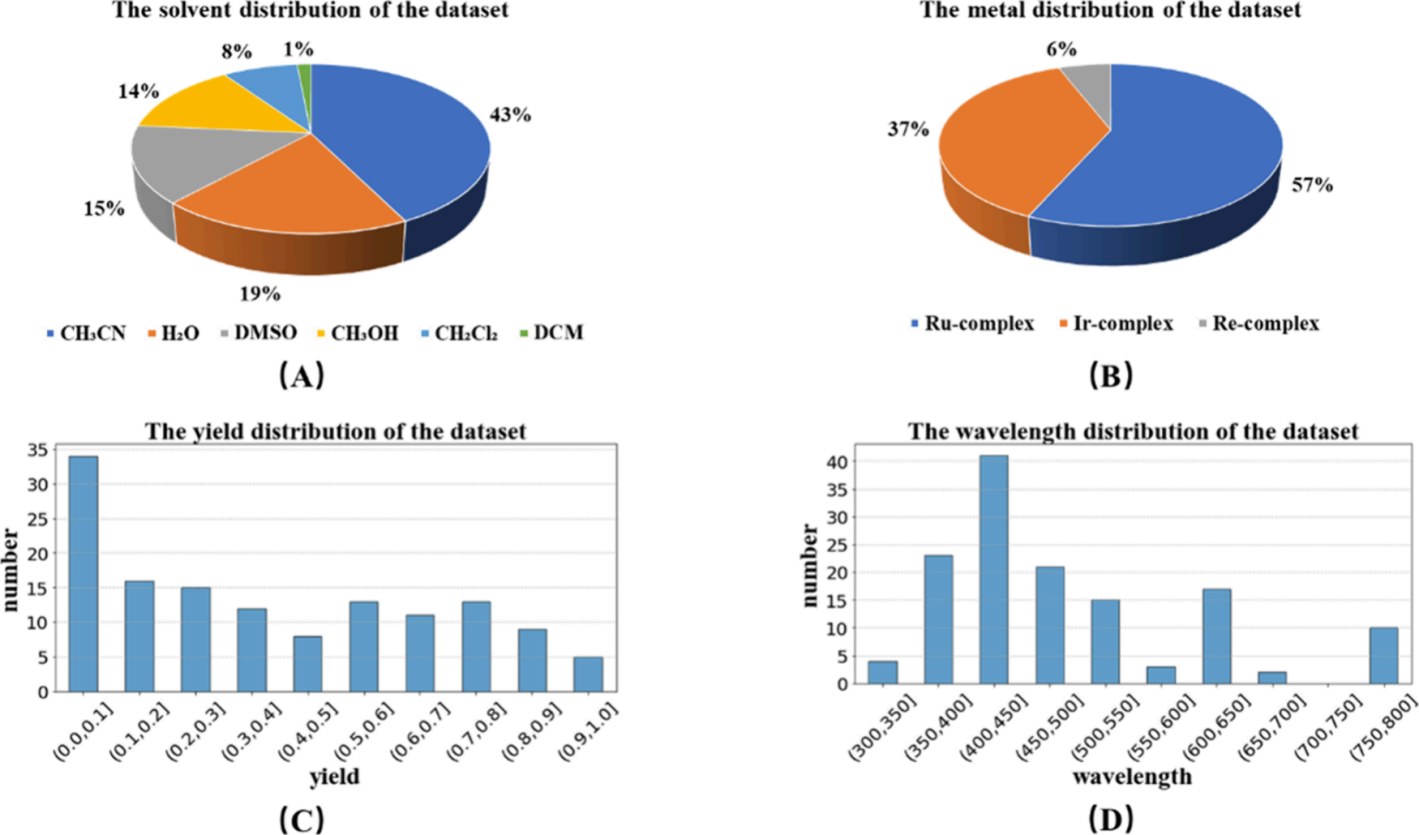
Distribution of the data set on (A) solvent, (B) metal center, (C) singlet oxygen quantum yield, and (D) irradiation wavelength.

### 2.2 Descriptor Acquisition

The descriptors used in this work consist of four kinds of descriptors for TMC photosensitizers. Quantum chemistry descriptors are the most important descriptors, which reflect the electron transfer information on S1 and T1 excited states and their differences calculated by time-dependent density functional theory (TD-DFT). Molecule structure descriptors include molecule size, charge, and structure on the photodynamic property. Metal-centered descriptors depict the impact of the metal center to distinguish the different kinds of TMC. External condition descriptors describe the impact of external conditions on the effect of the PDT process. The descriptors employ both implicit and explicit methods to describe the influence of the solvent. The implicit method incorporates the solvent’s effect into quantum chemistry descriptors through the CPCM and SMD solvent models used in DFT calculations. The explicit method, on the other hand, directly uses the static dielectric constant and dielectric constant at the infinite frequency of the solvent as external condition descriptors.

#### 2.2.1 Quantum Chemistry Descriptors

In the TD-DFT calculation, the excited state wave function is described by a linear combination of single excited configuration functions. Each configuration function has a coefficient *w* as excited configuration or *w*′ as deexcited configuration. First, hole distribution ρ^hole^ and electron distribution ρ^ele^ are defined as follows:

$$\rho^{\mathrm{hole}}(r)=\rho_{\mathrm{local}}^{\mathrm{hole}}(r)+\rho_{\mathrm{cross}}^{\mathrm{hole}}(r)$$ 1

$$\rho_{\mathrm{local}}^{\mathrm{hole}}(r)=\sum_{i\to a}(w_{i}^{a}{)}^{2}\varphi_{i}\varphi_{i}-\sum_{i\leftarrow a}(w_{i}^{\prime a}{)}^{2}\varphi_{i}\varphi_{i}$$ 2

$$\rho_{\mathrm{cross}}^{\mathrm{hole}}(r)=\sum_{i\to a}\sum_{j\neq i\to a}w_{i}^{a}w_{j}^{a}\varphi_{i}\varphi_{j}-\sum_{i\leftarrow a}\sum_{j\neq i\leftarrow a}w_{i}^{\prime a}w_{j}^{\prime a}\varphi_{i}\varphi_{j}$$ 3

$$\rho^{\mathrm{ele}}(r)=\rho_{\mathrm{local}}^{\mathrm{ele}}(r)+\rho_{\mathrm{cross}}^{\mathrm{ele}}(r)$$ 4

$$\rho_{\mathrm{local}}^{\mathrm{ele}}(r)=\sum_{i\to a}(w_{i}^{a}{)}^{2}\varphi_{a}\varphi_{a}-\sum_{i\leftarrow a}(w_{i}^{\prime a}{)}^{2}\varphi_{a}\varphi_{a}$$ 5

$$\rho_{\mathrm{cross}}^{\mathrm{ele}}(r)=\sum_{i\to a}\sum_{i\to b\neq a}w_{i}^{a}w_{i}^{b}\varphi_{a}\varphi_{b}-\sum_{i\leftarrow a}\sum_{i\leftarrow b\neq a}w_{i}^{\prime a}w_{i}^{\prime b}\varphi_{a}\varphi_{b}$$ 6

In eqs 1–6, *r* is the coordinate vector; φ is the orbital wave function; *i* or *j* is the occupied orbital, and *a* or *b* is the empty orbital. $\sum_{i\to a}$ means summation over each excited configuration and $\sum_{i\leftarrow a}$ means summation over each deexcited configuration, while *w*_*i*_^*a*^ is the coefficient of the excited configuration from occupied orbital *i* to empty orbital *a* and *w*_*i*_^′*a*^ is the coefficient of the deexcited configuration from empty orbital *a* to occupied orbital *i*. The hole distribution and electron distribution are divided into two parts: local term and cross term. The local term is generally dominant, reflecting the contribution of the configuration function itself, while the cross term reflects the impact of the coupling between the configuration functions on the hole and electron distribution. Then we adopt the following excitation descriptors:^46^

$$\mathrm{Sr}\;\mathrm{index}=\int \sqrt{\rho^{\mathrm{hole}}(r)\rho^{\mathrm{ele}}(r)}dr$$ 7

$$D\;\mathrm{index}=\sqrt{|X^{\mathrm{ele}}-X^{\mathrm{hole}}{|}^{2}+|Y^{\mathrm{ele}}-Y^{\mathrm{hole}}{|}^{2}+|Z^{\mathrm{ele}}-Z^{\mathrm{hole}}{|}^{2}}$$ 8

$$\Delta \sigma \;\mathrm{index}=|\sigma^{\mathrm{ele}}|-|\sigma^{\mathrm{hole}}|$$ 9

$$H_{\mathrm{CT}}=|H\times u_{\mathrm{CT}}|$$ 10

$$H\;\mathrm{index}=(|\sigma^{\mathrm{ele}}|+|\sigma^{\mathrm{hole}}|)/2$$ 11

$$t\;\mathrm{index}=D\;\mathrm{index}-H_{\mathrm{CT}}$$ 12

$$\mathrm{HDI}=100\times \sqrt{\int [\rho^{\mathrm{hole}}(r){]}^{2}dr}$$ 13

$$\mathrm{EDI}=100\times \sqrt{\int [\rho^{\mathrm{ele}}(r){]}^{2}dr}$$ 14

In these descriptors, the Sr index describes the degree of overlap between electron and hole distributions. The *D* index describes the distance between the mass centers of the electron and hole distributions, where *X* ^ele/hole^ is the *X* coordinate of the mass center of the electron/hole and *Y* ^ele/hole^, *Z* ^ele/hole^ stand for the other coordinate. The Δσ index describes the difference in the overall spatial distribution width of the electron and hole. **σ** ^**ele**^ and **σ** ^**hole**^ are the distribution breadth or dispersion degree of the electron and hole, and their *x*, *y*, and *z* components are the square mean root deviations of the electron and hole distribution with the *X*, *Y*, and *Z* coordinate of the mass center of the electron and hole calculated by eq 15. *H*_CT_ describes the average spread of electron and hole in the charge-transfer (CT) direction, where **H** is the sum of the average spread of electron and hole in *x*, *y*, and *z* direction, *s* calculated by eq 16 and **u**_**CT**_ is a unit vector in the CT direction. The *H* index describes the overall average width of the distribution of electrons and holes. The *T* index describes the degree of separation between electrons and holes. The hole delocalization index and electron delocalization index describe the uniformity of the distribution of holes and electrons.

$$\sigma_{\lambda }^{s}=\sqrt{\int (\lambda -\Gamma^{s}{)}^{2}\rho^{s}(r)dr},\;\lambda =\{x,y,z\},\;\Gamma =\{X,Y,Z\},\;s=\{\mathrm{hole},\mathrm{ele}\}$$ 15

$$H_{\lambda }=\frac{\sigma_{\lambda }^{\mathrm{ele}}+\sigma_{\lambda }^{\mathrm{hole}}}{2},\;\lambda =\{x,y,z\}$$ 16

Additionally, vertical ionization energy (VIE) and vertical electron affinity (VEA) are also candidate QCD; they are critically important for characterizing electron-transfer processes, which are fundamental to Type I PDT mechanisms.^47,48^ They are not calculated as the QCD in our model because they are not directly correlated with the singlet oxygen quantum yield for typical Type II systems.

First, DFT geometry optimizations were carried out using ORCA 5.0.4 ^49,50^ under the PBE0-D3 method^51^ and def2-SVP basis set;^52,53^ the solvation effect was considered using the CPCM solvent model;^54,55^ then use TD-DFT to complete S1 state and T1 state geometry optimization and calculation under PBE0-D3 method and def2-TZVP basis set^52,53,56^ with SMD solvent model^57^ to get excited state wave function information for subsequent descriptor calculations; finally, S1-T1 spin–orbit coupling (SOC) is calculated under the same calculation level as excited state calculation at optimized S1 state structure. All the following quantum chemistry descriptors of S1 (S-) and T1 (T-) states shown in Table 1 are calculated by the software Multiwfn 3.8 dev.^58^ The property differences (D-) between the S1 state and T1 state were also calculated as QCD. The PBE0 hybrid functional was selected because it has demonstrated accuracy similar to the B3LYP functional, which is one of the most widely used functionals. Moreover, the PBE0 functional is particularly well-suited for transition metal complexes, as it often provides better results in geometric optimization and energy calculation.^59,60^

**Table 1:** Meanings of Quantum Chemistry Descriptors

descriptors	meaning
*S/T/D-sr*	degree of overlap between electron and hole distributions of S1 state/of T1 state/of their difference
*S/T/D-d*	distance between the mass centers of the hole and electron distributions of the S1 state/of the T1 state/of their difference
S/T/D-sigma	difference in the overall spatial distribution width of the electron and hole of the S1 state/of the T1 state/of their difference
*S/T/D-hct*	average spread of the electron and hole in the CT direction of the S1 state/of the T1 state/of their difference
*S/T/D-h*	overall average width of the distribution of the electron and hole of the S1 state/of the T1 state/of their difference
*S/T/D-t*	degree of separation between the electron and hole of the S1 state/of the T1 state/of their difference
*S/T/D-hdi*	uniformity of the distribution of the hole of the S1 state/of the T1 state/of their difference
*S/T/D-edi*	uniformity of the distribution of electrons of the S1 state/of the T1 state/of their difference
*S/T/D-ee*	excitation energy of the S1 state/of the T1 state/difference between S1 and T1 state
*S/T/D-mlct*	metal to ligand charge transfer proportion of the S1 state/of the T1 state/of their difference
*S/T/D-tedm*	transition electric dipole moment of the S1 state/of the T1 state/of their difference
*S/T/D-tmdm*	transition magnetic dipole moment of the S1 state/of T1 state/of their difference
*soc*	spin–orbit coupling (SOC) matrix element between S1 and T1 states
*S1*	absorption wavelength of the S1 state calculated by DFT
*fosc1*	oscillator strengths of the S1 state via transition electric dipole moments
*fosc2*	oscillator strengths of the S1 state via transition velocity dipole moments

#### 2.2.2 Molecule Structure Descriptors

Molecule structure descriptors describe the impact of molecule size, charge, and structure on the photodynamic property including the charge of entire complex cation, the charge of all ligand, the charge of connected atom with metal center, the number of atoms, relative molecular mass, and the number of important functional groups (if a functional group contains several smaller functional groups, the largest functional group is counted). Take two complexes shown in Table S1 as an example. For complex 1, the entire complex cation has a valence of +2, so *nc* = 2, all three ligands are electrically neutral, so *lc* = 0; among the 6 atoms connected to ruthenium, the oxygen carries a unit of negative charge, so *cc* = −1; the Ru in the complex has a valence of +2, so *mc* = 2. By the same reasoning, for complex 17, *nc* = 2, *lc* = 0, *cc* = 0, and *mc* = 2. The detailed meanings are shown in Table 2.

**Table 2:** Meanings of Molecular Structure Descriptors

descriptors	meaning
*nc*	net charge of the entire complex cation
*lc*	charge of all ligands
*cc*	charge of connected atoms with a metal center
*an*	number of atoms
*mw*	relative molecular mass
*n-X*	number of halogens
*n-COO*	number of ester groups
*n-CO*	number of carbonyl groups
*n-CHO*	number of aldehyde groups
*n-CONH*	number of peptide bonds
*n–OH*	number of hydroxyl groups
*n-NH2*	number of amino groups
*n-S*	number of sulfur atoms
*n-O*	number of oxygen atoms
*n-CN*	number of cyan groups
n-bodipy	number of dipyrromethene boron difluorides
*n-py*	number of pyridines
*n-ph*	number of phenyl groups
*n-pyra*	number of pyrazines
*n-pyrr*	number of pyrroles
*n-r6*	number of six-membered rings
*n-r5*	number of five-membered rings
*n-db*	number of double bonds
*n-tb*	number of triple bonds

#### 2.2.3 Metal-Centered Descriptors

Metal-centered descriptors distinguish the impact of different transition metals as center on TMC properties by describing the number of metal center, the charge of metal center (*mc*), the period which the metal element is located in (*cp*), and the outer electron configuration of metal atoms (*cs, cd, cf* for s-, d- and f- electron count). The outer electron configuration of metal atoms is based solely on the intrinsic properties of the free metal atom (prior to coordination). For example, Ru is in period 5, so *cp* = 5; Ru has the configuration [Kr]­4d^7^5s^1^, so *cs* = 1, *cd* = 7, and *cf* = 0. The detailed meanings are shown in Table 3.

**Table 3:** Meanings of Metal-Centered Descriptors

descriptors	meaning
*cn*	number of metal centers
*cp*	period in which the central metal element is located
*mc*	charge of the metal center
*cf*	number of electrons in the outermost f orbital of the central metal
*cd*	number of electrons in the outermost d orbital of the central metal
*cs*	number of electrons in the outermost s orbital of the central metal

#### 2.2.4 External Condition Descriptors

External condition descriptors describe the impact of external condition on the effect of PDT process including the static dielectric constant, dielectric constant at infinite frequency of the solvent, and irradiation wavelengths. The detailed meanings are shown in Table 4.

**Table 4:** Meanings of External Condition Descriptors

descriptors	meaning
*eps*	static dielectric constant of the solvent
*epsinf*	dielectric constant at the infinite frequency of the solvent
*wl*	number of electrons in the outermost s orbital of the central metal

### 2.3 Machine Learning Models

The data set was randomly divided; the training set accounts for 90% (122 data points), and the test set accounts for 10% (14 data points). Leave-one-out (LOO) cross-validation was used on the training set to test the stability of the model. The trained models are also tested on the external test set to obtain their generalization ability. All the input descriptors are normalized by eq 17 (soc is normalized after the logarithm is taken).

$$X_{\mathrm{normalized}}=\frac{X-X_{\min}}{X_{\max}-X_{\min}}$$ 17

Six candidate single-ML models are first utilized for the prediction of small data set TMC photosensitizers: support vector regression (SVR), kernel ridge regression (KRR), Gaussian process regression (GPR), eXtreme Gradient Boosting regression (XGBoost), random forest regression (RFR), and k-neighbor regression (KNR). These models are first trained on all descriptors to achieve the descriptors' importance rank through SHAP analysis, and then the first 30–50 descriptor groups (at intervals of 5) are tested as the model input and retrained these models to get the best descriptor group. To further improve the prediction accuracy and generalization ability of the single-ML model, two hybrid models are proposed: the delta-learning model (DLM) and the Mixture-of-Experts model (MoE). The process of the delta-learning model is shown in Figure 4. The first model is used to predict the target value, and the next model is used to predict the error (delta) of the real value and predicted value of the previous model as an amendment item, and so on. Thus, the final predicted value is the predicted value by the first model compared to the predicted errors by all subsequent models. MoE uses multiple single-ML models as expert models to predict the target value simultaneously, as shown in Figure 5. The final predicted value of the MoE model is the weighted average of the predicted values of multiple machine learning models. All the model parameters above are optimized by the optuna library in Python 3.11, and the hyperparameters optimized for these models are shown in Table 5.

**Figure 4:**
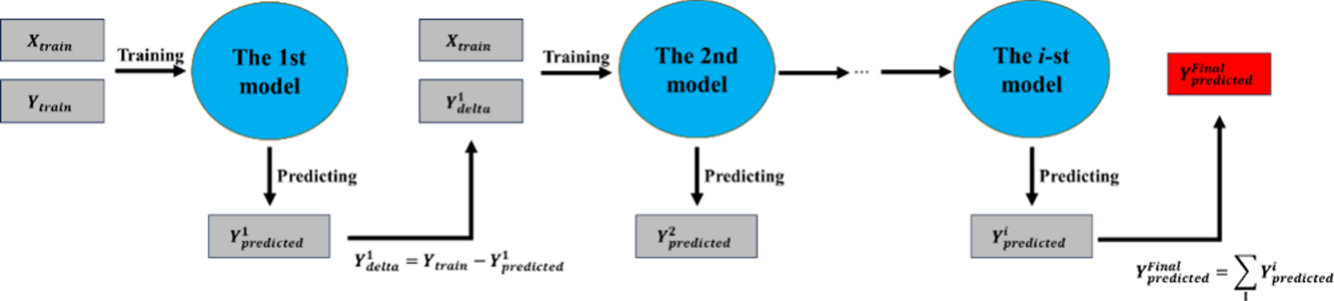
Process of the delta-learning model.

**Figure 5:**
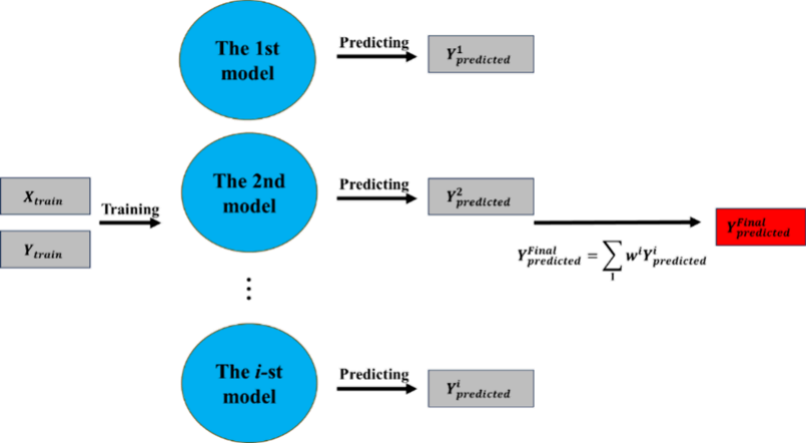
Process of the Mixture-of-Experts model.

**Table 5:** Hyperparameters Optimized of Single-ML Models

models	parameters optimized
SVR	penalty coefficient, tolerance, kernel type
KRR	regularization parameter, hyperparameter of Gaussian kernel
GPR	noise variance, kernel length scale, number of optimizers
XGBoost	max depth of the tree, learning rate, number of estimators, sample ratio and feature ratio of each tree
RFR	number of decision tree; number of features considered per branch; max depth of the decision tree
KNR	number of neighbors, distance measurement parameters, weight allocation strategies, nearest neighbor search algorithms

The target variable for all machine learning models developed in this work is the experimentally measured Φ_Δ_. Because the machine learning models are inherently nonlinear and very complex in explicit form, the subsequent SHAP analysis is employed to show descriptors contribution to the predicted Φ_Δ_ which could provide strong interpretability of the proposed model.

## 3 Results and Discussion

In this section, we present the result of the descriptor filter and the training result with the best descriptor group of single-ML models. We use SHAP analysis to determine the importance of descriptors and choose the most important ones. Then, we show the performance of two hybrid models to find the best model to predict the Φ_Δ_ of TMC photosensitizers. Finally, we compare the generalized metal model trained on three six-coordination TMCs with the specialized metal model trained on a specific TMC.

### 3.1 Single-ML Models

In order to verify the importance of different kinds of descriptors, we first compared these models using all descriptors against models where one kind of descriptor was removed at a time. The key finding is that the removal of QCD led to the most significant drop in model performance (the *R* ^2^ of the external test set decreased from 0.830 to 0.051 in SVR, from 0.747 to 0.214 in KRR, and from 0.451 to −0.142 in GPR) as shown in Table S3. This demonstrates that the QCD provides unique and critical information that cannot be compensated for by the other descriptors. After the descriptor filter, the best models are the SVR model and the KRR model, followed by GPR, as shown in Tables 6 and S4. These models with the best descriptor groups show a good regression effect, generalization ability, as shown in Table 7 and Figure 6. The stability of these three models is not very well because of the lack of data set, the complexity of the mechanism of the photodynamic therapy process, and the different environmental influences during the Φ_Δ_ testing process. The other three single-ML models do not satisfy the conditions of the QSPR model (*Q* ^2^ ≥ 0.6 in cross-validation and *R* ^2^ ≥ 0.6 in the external test set).^61^ The superior performance of the kernel-based models (SVR, KRR, and GPR) over tree-based models (RFR and XGBoost) can be attributed to the nature of our feature space. Our descriptor set primarily consists of continuous, normalized quantum-chemical, and structural properties. Kernel methods are particularly adept at modeling complex, nonlinear relationships in such continuous feature spaces by implicitly mapping them into higher-dimensional spaces where linear relationships may be found. In contrast, tree-based models, which rely on axis-aligned splits, often perform exceptionally well with highly dimensional, sparse data such as molecular fingerprints. The descriptors' importance ranking by SHAP analysis of six single-ML models with all descriptors is shown in Figures S1–S6.

**Table 6:** *R* ^2^(*Q* ^2^) Result of the SVR Model and KRR Model in a Descriptor Filter

model	*R* ^2^(*Q* ^2^)	30 descriptors	35 descriptors	40 descriptors	45 descriptors	50 descriptors	all descriptors
SVR	training set	0.970	0.957	0.992	0.992	0.990	0.990
	test set	0.948	0.947	0.940	0.927	0.940	0.935
	external test set	0.681	0.603	0.741	0.815	0.772	0.830
	LOO cross-validation	0.568	0.618	0.619	0.622	0.626	0.579
KRR	training set	0.991	0.998	0.996	0.997	0.996	0.993
	test set	0.942	0.914	0.884	0.903	0.924	0.944
	external test set	0.713	0.663	0.700	0.753	0.729	0.747
	LOO cross-validation	0.594	0.608	0.616	0.635	0.534	0.593

**Table 7:** Performance of Three Best Models with Filtered Descriptors

model		*R* ^2^(*Q* ^2^)	MaxAE	MAE	MSE
SVR (45 filtered descriptors)	training set	0.992	0.091	0.024	0.001
	test set	0.927	0.130	0.049	0.004
	external test set	0.815	0.254	0.091	0.016
	LOO cross-validation	0.622	0.631	0.122	0.033
KRR (45 filtered descriptors)	training set	0.997	0.055	0.011	0.0003
	test set	0.903	0.157	0.061	0.005
	external test set	0.753	0.296	0.115	0.021
	LOO cross-validation	0.635	0.610	0.119	0.032
GPR (40 filtered descriptors)	training set	0.989	0.117	0.019	0.001
	test set	0.866	0.261	0.056	0.007
	external test set	0.701	0.303	0.125	0.025
	LOO cross-validation	0.647	0.556	0.122	0.031

**Figure 6:**
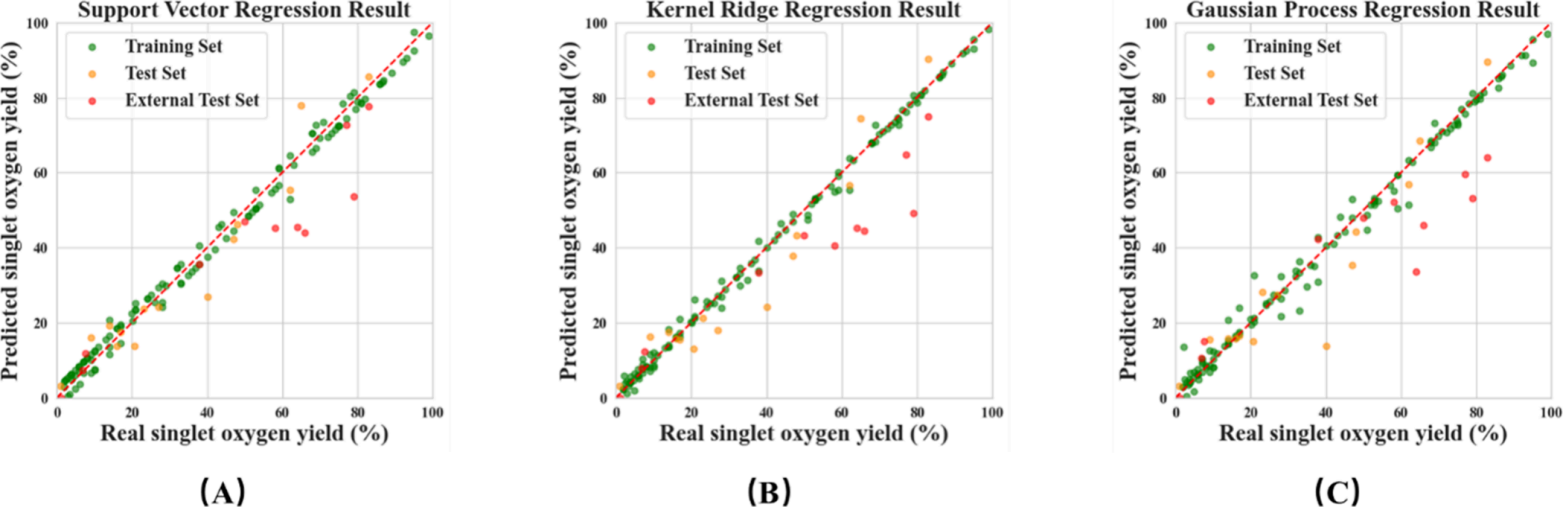
Performance of (A) SVR model, (B) KRR model, and (C) GPR model on the training set, test set, and external test set.

The filtered descriptors contribution is sorted by SHAP analysis,^62^ shown in Figures S7–S9. Among the top 15 descriptors of the three models, 12 are the same, indicating that these descriptors can well describe the influence factors of the photodynamic therapy process, and these models can also recognize these factors as shown in Figure 7A. QCD has a major influence on the descriptors, which indicates that these models have strong interpretability of the PDT mechanism as shown in Figure 7B. The excitation energy of S1 state (*S-ee*) and T1 state (*T-ee*) as QCD are the two most important descriptors, which influence the Φ_Δ_ by Δ*E*_st_ in ISC process. The notable importance of molecule structural descriptors such as relative molecular mass (*mw*) and number of atoms (*an*), while not directly involved in the photophysical process, can be interpreted as a consequence of the data set’s composition. We posit that the relative molecular mass acts not as a direct causal factor but rather as a proxy variable for molecular complexity, which is a key outcome of successful ligand engineering. To achieve high singlet oxygen quantum yields, sophisticated ligand modifications, such as extending π-conjugation, are typically employed. These modifications inevitably increase the molecular mass and atom number. Consequently, the model identifies a correlation wherein higher-performing molecules tend to possess more complex, and therefore heavier, architectures. This insight underscores that the model learns from the patterns of successful design presented in the literature.

**Figure 7:**
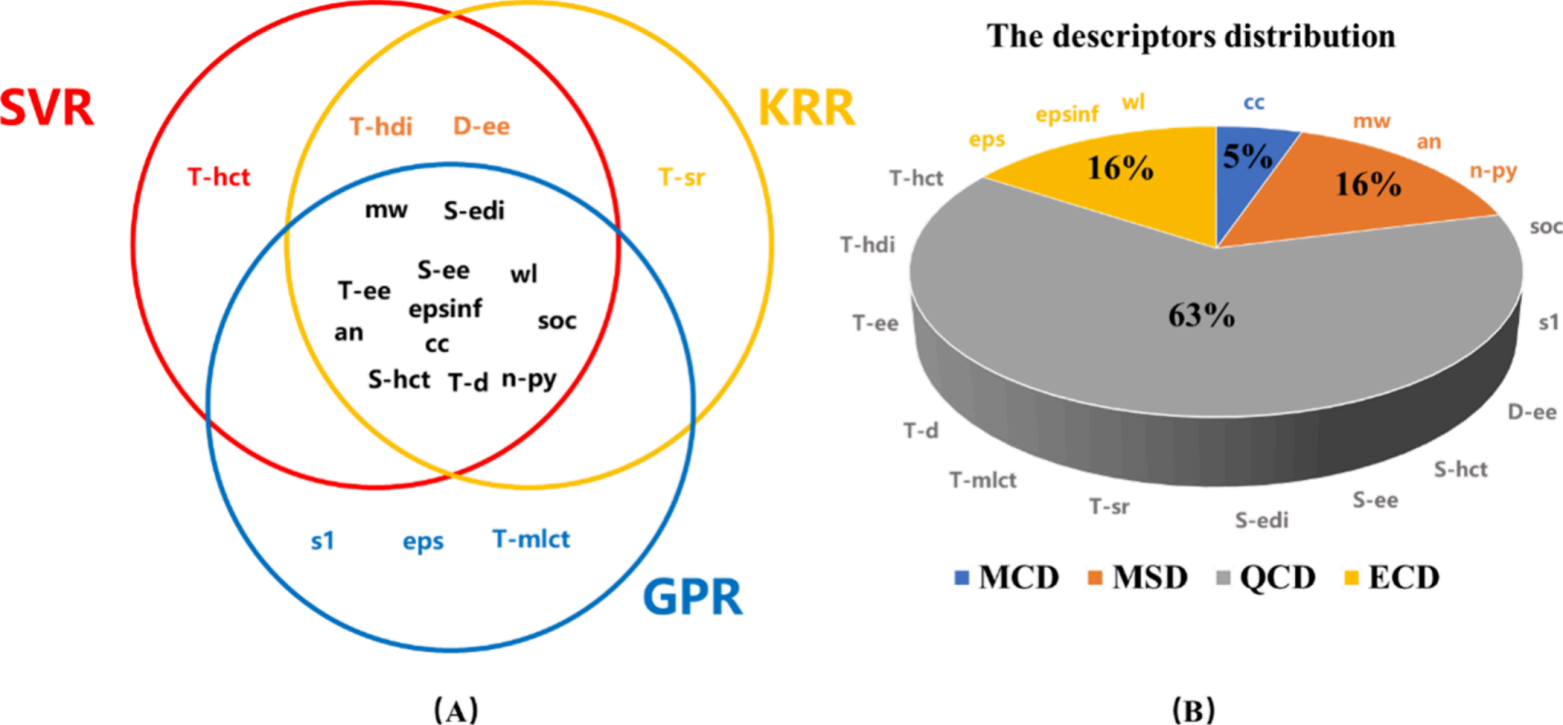
Modeling descriptor results: (A) distribution in 15 top descriptors of SVR, KRR, and GPR model by SHAP analysis separately; and the (B) type they belong to.

### 3.2 Hybrid-ML Models

#### 3.2.1 Delta-Learning Model

We use the delta-learning model with bilayers in this section. We selected the top-performing model, SVR, as the base model to provide a strong initial estimate. For the critical delta model, which must capture the complex pattern of errors, we chose the second-best performer, KRR. Compared with the SVR and KRR models, which constitute the delta-learning model, it not only shows better fitting effects on the training set and test set but also has better generalization ability on the external test set, as shown in Table 8 and Figure 8A. This indicates that the delta-learning model can correct the results of the single-ML model by further predicting the residual, thereby improving its generalization ability.

**Table 8:** Performance of DLM and MoE

model		*R* ^2^(*Q* ^2^)	MaxAE	MAE	MSE
DLM	training set	1.000	0.031	0.004	10^–5^
	test set	0.928	0.132	0.051	0.004
	external test set	0.820	0.256	0.091	0.015
	LOO cross-validation	0.625	0.628	0.120	0.032
mixture-of-SVR-KRR-GPR model (MoE1)	training set	0.990	0.067	0.025	0.001
	test set	0.922	0.132	0.055	0.004
	external test set	0.870	0.211	0.083	0.011
	LOO cross-validation	0.657	0.637	0.117	0.030
mixture-of-SVR-KRR model (MoE2)	training set	0.992	0.092	0.024	0.001
	test set	0.927	0.142	0.051	0.004
	external test set	0.840	0.234	0.085	0.014
	LOO cross-validation	0.621	0.660	0.120	0.033

**Figure 8:**
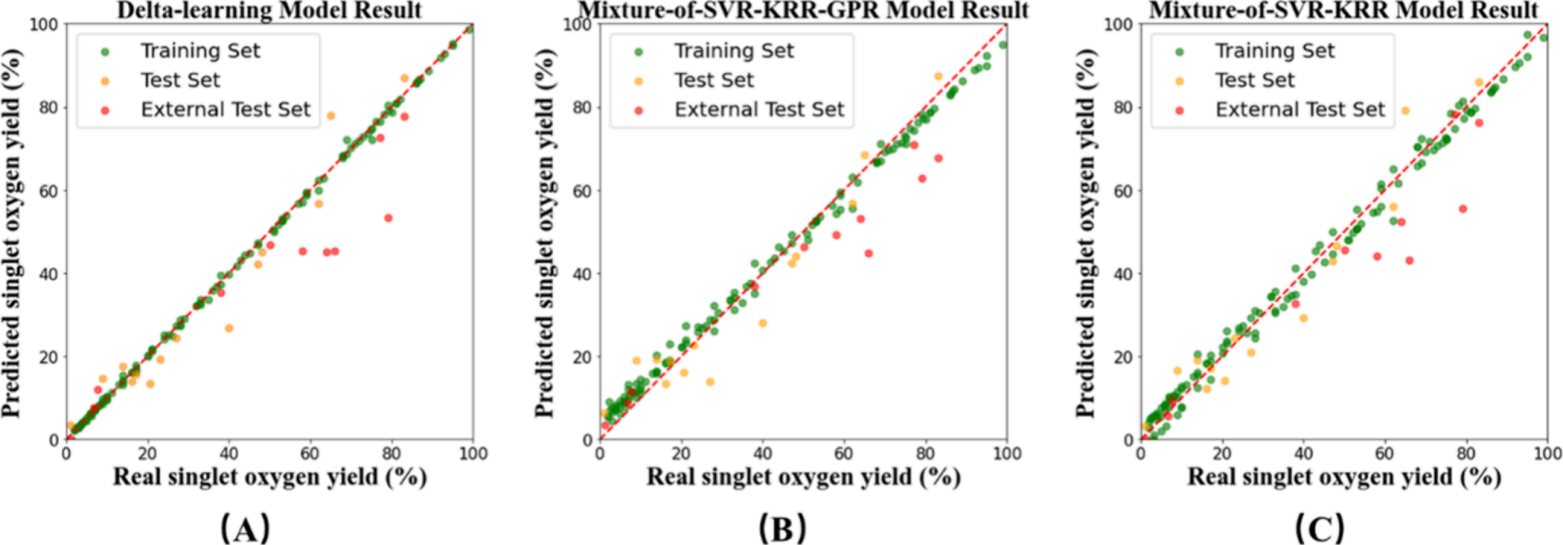
Performance of (A) DLM, (B) mixture-of-SVR-KRR-GPR model, and (C) mixture-of-SVR-KRR model on training set, test set, and external test set.

#### 3.2.2 Mixture-of-Experts Model

In this section, we designed two variants to systematically evaluate the impact of expert composition: MoE1 (3 Experts), which incorporates all three kernel-based models (SVR, KRR, and GPR), and MoE2 (2 Experts), which incorporates only the top two models (SVR and KRR). This comparative design allowed us to test whether the performance of the MoE model is optimal with a focused set of the best experts or a broader ensemble. The result shows that the MoE model also shows better fitting effects on the training set and test set and better generalization ability on the external test set, as shown in Table 8 and Figure 8B,C. This indicates that the MoE model balances the errors of each single-ML model and enhances the prediction ability of the model.

Judging from the performances of the training set and the test set, all three hybrid models have shown improved fitting effects. Judging from the performance of the external test set, among the three hybrid models, the best-performing model is the MoE1 model, followed by the MoE2 model and the delta-learning model. Thus, it is observed that hybrid models can improve the prediction accuracy and generalization ability of single-ML models for TMC photosensitizing properties.

### 3.3 Comparison with the Specialized Metal Model

In this section, we retrain the two kinds of hybrid models: the delta-learning model (DLM) and the MoE1 model from scratch on Ru-complex and Ir-complex photosensitizers, respectively (first train the single-ML model and get filtered descriptor groups, then optimize and retrain these two models). The result is compared with the performance of these two models trained in Section 2.3 on all TMC photosensitizers data sets to test whether the generalized metal model trained on three six-coordination TMC can replace the specialized metal model trained on TMC of a given metal center. The Ru-complex data set consists of 77 data points in the internal data set (used for training set/test set with a 9:1 random split) and 5 data points in the external test set. The Ir-complex data set consists of 51 data points in the internal data set and 5 data points in the external test set.

The generalized metal DLM performed slightly better on the Ru-complex data set compared to the model trained on all metal complexes (with the upper and lower quartiles error closer to 0), but slightly worse on the external test set, though the difference was minor, as shown in Figure 9. It also performed marginally better on the Ir-complex data set (with a smaller negative maximum error) and performed basically the same on the external test set. This indicates that the generalized metal DLM has strong universality and can replace the specialized metal DLM trained on Ru-complexes. The detailed result of DLM comparison is shown in Table S5.

**Figure 9:**
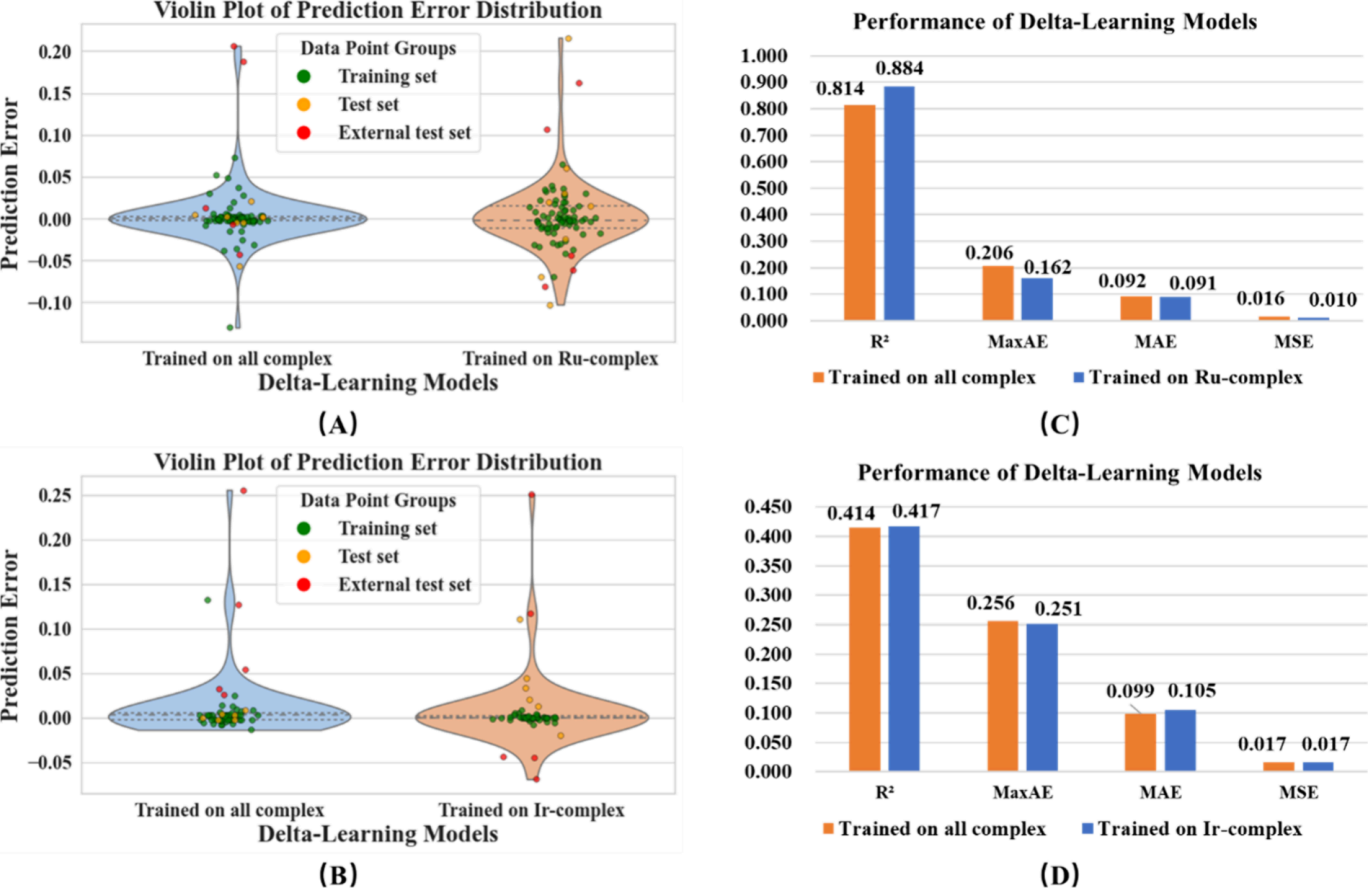
Violin plots of error distribution on (A) Ru-complex, (B) Ir-complex and the result comparisons of external test set on (C) Ru-complex, and (D) Ir-complex of DLM.

The performance of both DLM and the following MoE model on the external test set of the Ir-complex data set is poorer compared to that of the Ru-complex data set. The possible reasons are as follows: (I) The smaller data set for Ir-complexes might contribute to the observed difference. The model may have learned more robust patterns for Ru-complexes due to their larger representation, leading to more confident and accurate predictions for this class; (II) For many Ru-complexes, the dominant route for singlet oxygen generation involves the intersystem crossing (ISC) between the S1 and T1 state. In contrast, Ir-complexes, with their stronger spin–orbit coupling, may exhibit more complex excited-state dynamics. The generation of singlet oxygen may involve energy transfer from other triplet states (e.g., T2) or proceed through mixed triplet state character. Our current QCD, which focuses on S1 and T1 states, may not fully capture the critical energetics and dynamics associated with these alternative pathways, leading to reduced predictive accuracy for Ir-complexes.

In terms of the prediction of Ru-complexes, the generalized metal MoE1 model performs slightly worse (with a wider distribution of prediction errors) compared to the specialized metal MoE1 model, and its performance on the external test set is also slightly lower, as shown in Figure 10. In terms of the prediction of Ir-complexes, the generalized metal MoE1 model performs slightly better than the specialized metal MoE1 model (with lower maximum positive and negative errors), and their performance on the external test set is basically the same. This indicates that the MoE model also has strong universality and can be used to predict the properties of various TMC photosensitizers. Compared with DLM, the MoE model achieved better performance on both the external test sets of Ru-complexes and Ir-complexes. This might be because the MOE model using multiple monolayer models is more likely to learn patterns from small data sets than the DLM using a bilayer model. The detailed result of the MoE1 model comparison is shown in Table S6.

**Figure 10:**
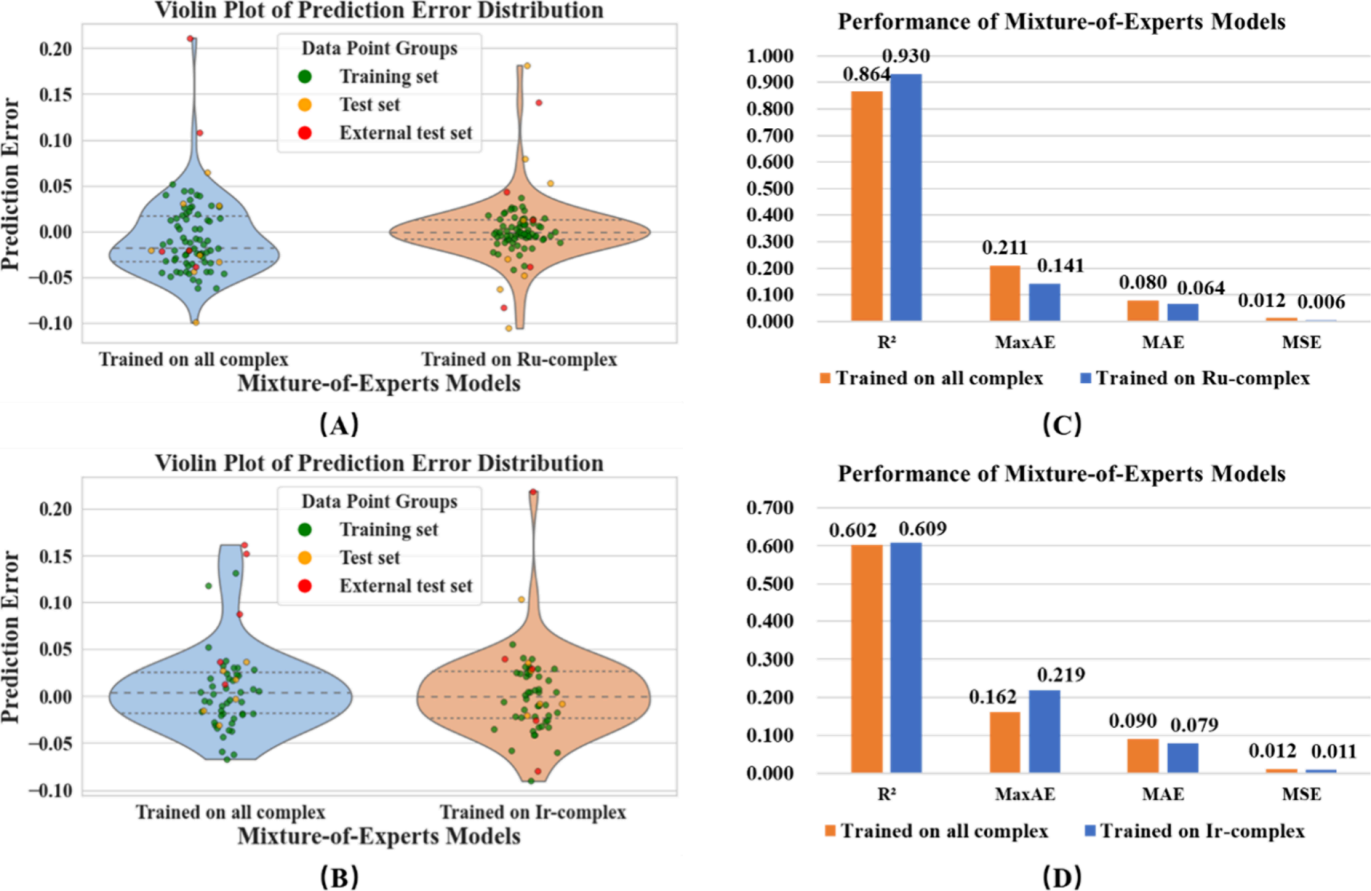
Violin plots of error distribution on (A) Ru-complex, (B) Ir-complex and the result comparisons of external test set on (C) Ru-complex, and (D) Ir-complex of the MoE1 model.

## 4 Conclusions

Transition metal complexes are potential photosensitizer candidates in PDT for their high singlet oxygen quantum yield (Φ_Δ_) and water solubility, but the synthesis of photosensitizers and the experimental determination of Φ_Δ_ are both time-intensive and laborious processes. Traditional structure descriptors for machine learning models, such as SMILES, can hardly capture all the information of TMC, and the lack of data makes it difficult to use deep learning models. In this work, we propose a DFT-ML modeling approach to predict the photosensitizing properties of TMC. The excited state descriptors are calculated by DFT, and ML models are built using them together with other descriptors to characterize the structure and charge transition process under light irradiation of TMC photosensitizers. Six single-ML models and two kinds of hybrid-ML models are proposed based on these descriptors and their performance on the Φ_Δ_ prediction is tested.

The best descriptor groups are filtered to optimize single-ML models, respectively, and the best single models are then utilized to build hybrid models. The results show SVR and KRR provide good predictions on the test set (*R* ^2^ > 0.9) and external test set (*R* ^2^ > 0.7) in single-ML models; while DLM and MoE models can further improve the prediction effect (*R* ^2^ up to 0.87 on the external test set). The comparison with the same hybrid-ML model trained on a specialized metal complex indicates that the proposed models also have strong universality (Δ*R* ^2^ < 0.1 on the external test set between the generalized metal model and the specialized metal model) and can be used to predict the properties of various TMC photosensitizers. The subsequent SHAP analysis provides strong interpretability of the PDT mechanism. The excitation energy of the S1 state and the T1 state are the two most important descriptors, while relative molecular mass and dielectric constant at infinite frequency of the solvent also have an outstanding impact. These results demonstrate that the excited state descriptors have a good effect in predicting PDT process properties, and the hybrid-ML model with these descriptors can provide accurate predictions on photosensitizing properties based on a small data set of TMCs. Thus, the proposed approach could be a useful addition and theoretical guidance as a screening step prior to experiments of organic synthesis and photosensitivity testing.

This approach can filter out a large proportion of promising but low-performing candidates in the computational stage. By reducing the number of compounds that require synthesis, the proposed model can (I) significantly decrease the consumption of valuable and expensive metal precursors, ligands, and other chemicals, (II) save weeks or months of synthetic and characterization labor, and (III) allow researchers to focus their experimental efforts on the most promising leads, thereby increasing the efficiency and success rate of the discovery pipeline. However, one should notice that the scarcity of experimentally measured Φ_Δ_ for TMC is a fundamental constraint in the field, directly leading to our small data set. In the future, we will work collaboratively to expand the data set by incorporating photosensitizers based on other metals (such as Pd, Pt, Zn, etc.), leading to a more robust and universally applicable model.

## Supplementary Material





## Notes

Detailed calculated descriptors input used for model training (XLSX)
Detailed information on TMC photosensitizers used to construct the data set and external test set, result of the GPR model, XGBoost model, RFR model, and KNR model in a descriptor filter, result of hybrid model comparison on specialized TMC photosensitizers, result of SHAP analysis, and optimized hyperparameters of the machine learning models (PDF)

## Abbreviations

TMC: transition metal complex
PS: photosensitizer
DFT: density functional theory
TD-DFT: time-dependent density functional theory
ML: machine learning
QSPR: quantitative structure–property relationship
PDT: photodynamic therapy
ROS: reactive oxygen species
ISC: intersystem crossing
CT: charge-transfer
QCD: quantum chemistry descriptor
MSD: molecule structure descriptor
MCD: metal-centered descriptor
ECD: external condition descriptor
LOO: leave-one-out
SVR: support vector regression
KRR: kernel ridge regression
GPR: Gaussian process regression
XGBoost: extreme gradient boosting regression
RFR: random forest regression
KNR: k-neighbor regression
DLM: delta-learning model
MoE: Mixture-of-Experts
