# Supplementary material for: DFT-ML-Based Property Prediction of Transition Metal Complex Photosensitizers for Photodynamic Therapy
Source: ACS Omega. 2025 Oct 31;10(44):53447–59. doi: 10.1021/acsomega.5c08727 (PMC12613122; doi:10.1021/acsomega.5c08727)
Supplement: Supplementary file 2 [file ao5c08727_si_002.pdf]

Supporting Information

DFT-ML based Property Prediction of Transition Metal Complex Photosensitizers for Photodynamic Therapy

Jingxing Gao, Yachao Dong*, Tian Qiu, Wen Sun*, Jian Du

School of Chemical Engineering, Dalian University of Technology,

Dalian 116024, China

*Email of corresponding authors:

yachaodong@dlut.edu.cn (Yachao Dong);

sunwen@dlut.edu.cn (Wen Sun)

Contents

Table S1. The detail of TMC photosensitizers dataset.......... 3

Table S2. The detail of TMC photosensitizers data in external test set.......... 20

Table S3. The performances of single-ML models with a kind of descriptors removed... 23

Table S4. the R2(Q2) result of GPR, XGBoost, RFR and KNR in descriptors filter 24

Table S5. The delta-learning model comparison on specialized TMC photosensitizers... 25

Table S6. The Mixture-of-Experts model comparison on specialized TMC photosensitizers.......... 25

Figure S1. SHAP analysis of SVR model with all descriptors.......... 26

Figure S2. SHAP analysis of KRR model with all descriptors 27

Figure S3. SHAP analysis of GPR model with all descriptors.......... 28

Figure S4. SHAP analysis of XGBoost model with all descriptors.......... 29

Figure S5. SHAP analysis of RFR model with all descriptors.......... 30

Figure S6. SHAP analysis of KNR model with all descriptors 31

Figure S7. SHAP analysis of SVR model with filtered descriptors.......... 32

Figure S8. SHAP analysis of KRR model with filtered descriptors.......... 33

Figure S9. SHAP analysis of GPR model with filtered descriptors 34

The optimized hyperparameters of machine learning models 35

Table S1. The detail of TMC photosensitizers dataset

Number	Structure	Solvent	Irradiation wavelength	Singlet oxygen quantum yield	Reference
1	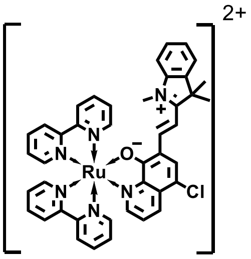	CH_3CN	650nm	0.033	1
2	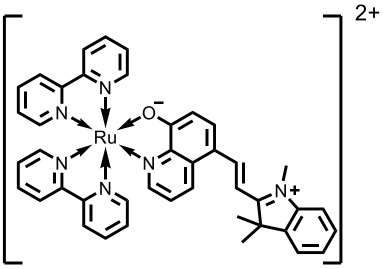	CH_3CN	650nm	0.058	1
3	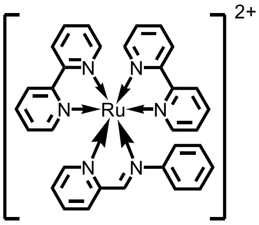	H_2O	465nm	0.025	2
4	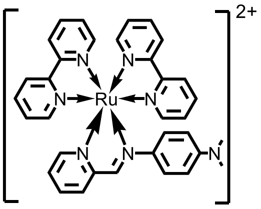	H_2O	465nm	0.036	2

5	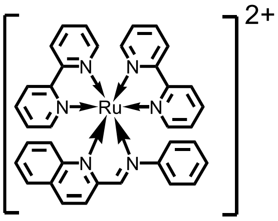	H_2O	465nm	0.061	2
6	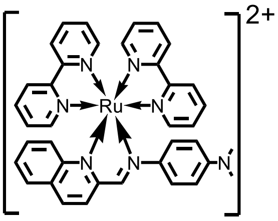	H_2O	465nm	0.046	2
7	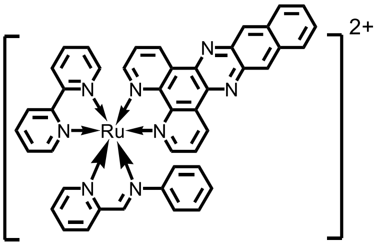	H_2O	465nm	0.27	2
8	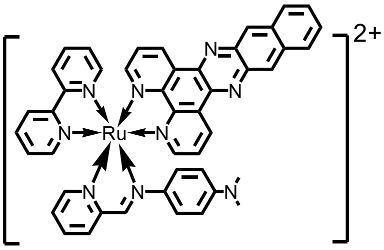	H_2O	465nm	0.33	2
9	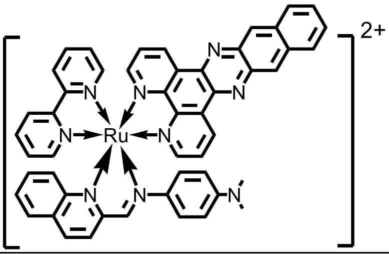	H_2O	465nm	0.36	2
10	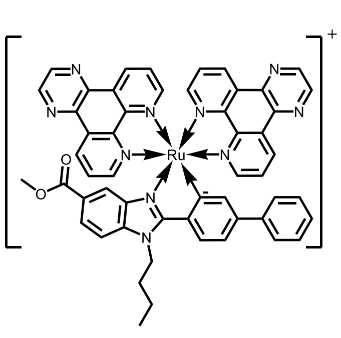	CH_3CN	355nm	0.07	3
		CH_3CN	532nm	0.09	3
		CH_2Cl_2	355nm	0.07	3
		CH_2Cl_2	532nm	0.08	3

11	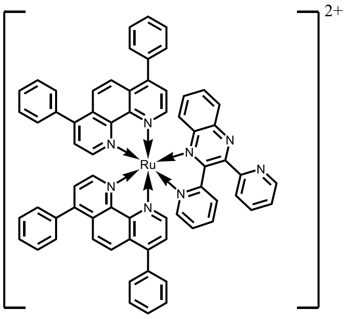	H_2O	530nm	0.38	4
		H_2O	632nm	0.23	4
12	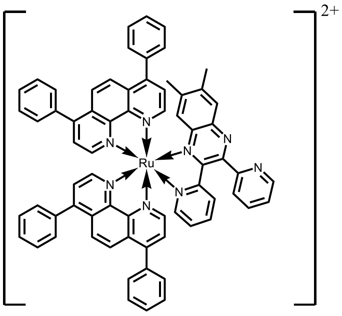	H_2O	528nm	0.62	4
		H_2O	632nm	0.33	4
13	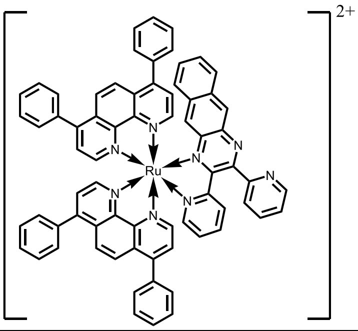	H_2O	570nm	0.35	4
		H_2O	632nm	0.14	4
14	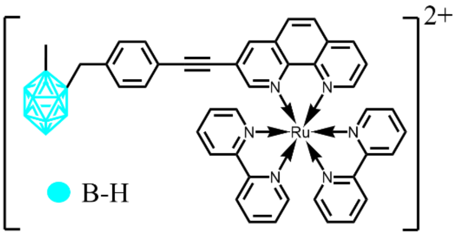	CH_3CN	355nm	0.52	5
15	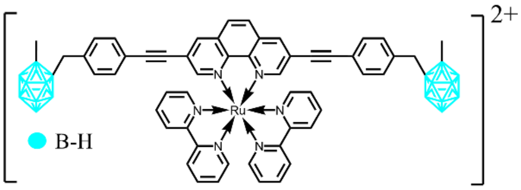	CH_3CN	355nm	0.2	5

16	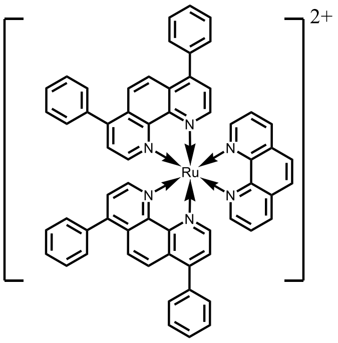	DMSO	450nm	0.71	6
17	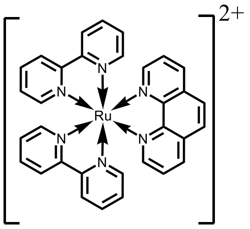	DMSO	450nm	0.47	6
18	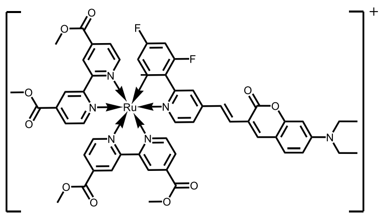	CH_3CN	475nm	0.14	7
19	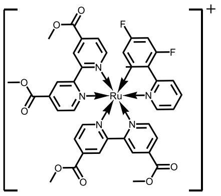	CH_3CN	475nm	0.16	7
20	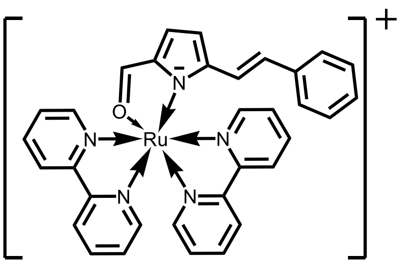	CH_3CN	514nm	0.13	8
21	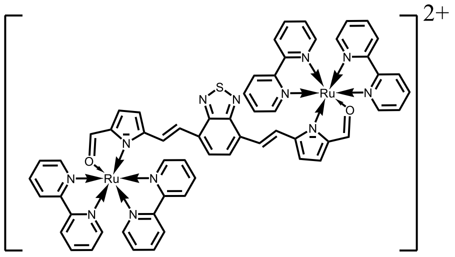	CH_3CN	602nm	0.32	8

22	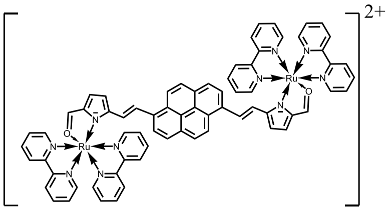	CH_3CN	508nm	0.68	8
23	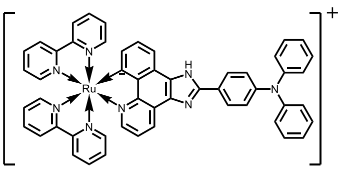	CH_3OH	475nm	0.09	9
24	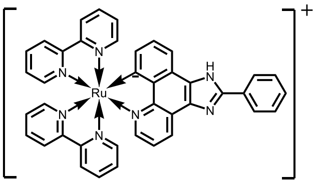	CH_3OH	475nm	0.1	9
25	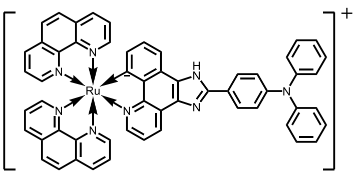	CH_3OH	475nm	0.28	9
26	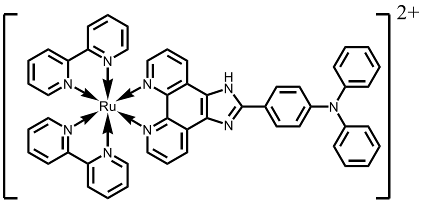	CH_3OH	475nm	0.16	9
27	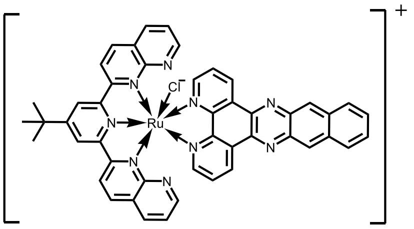	CH_3CN	630nm	0.04	10
		CH_3CN	753nm	0.02	10
28	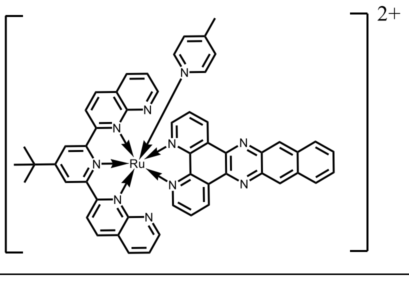	CH_3CN	630nm	0.93	10
		CH_3CN	753nm	0.86	10

29	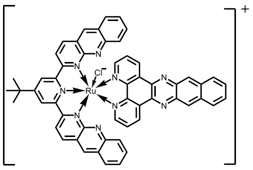	CH_3CN	464nm	0.05	10
		CH_3CN	630nm	0.01	10
		CH_3CN	753nm	0.03	10
30	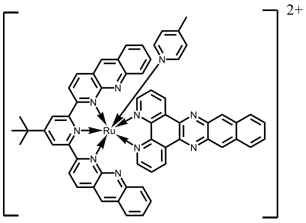	CH_3CN	455nm	0.14	10
		CH_3CN	630nm	0.09	10
		CH_3CN	753nm	0.03	10
31	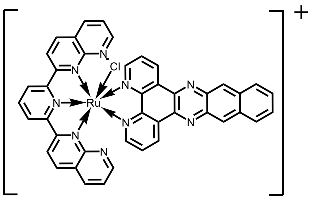	CH_3CN	464nm	0.28	10
		CH_3CN	630nm	0.09	10
		CH_3CN	753nm	0.07	10
32	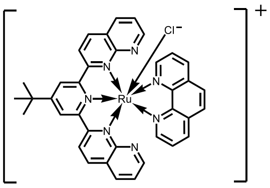	CH_3CN	455nm	0.06	10
		CH_3CN	630nm	0.04	10
		CH_3CN	753nm	0.05	10
33	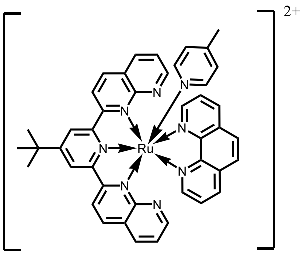	CH_3CN	455nm	0.17	10
		CH_3CN	630nm	0.1	10
		CH_3CN	753nm	0.1	10
34	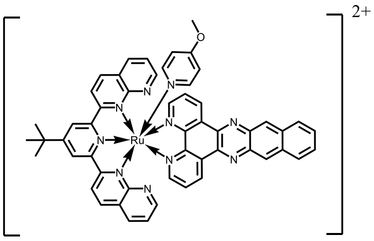	CH_3CN	630nm	0.69	10
		CH_3CN	632nm	0.75	10
		CH_3CN	753nm	0.69	10

35	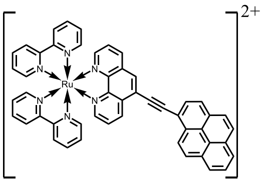	CH_3CN	412nm	0.68	11
36	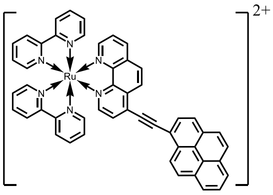	CH_3CN	482nm	0.87	11
37	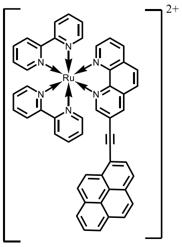	CH_3CN	414nm	0.65	11
38	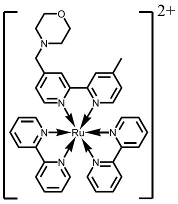	CH_3OH	450nm	0.509	12
39	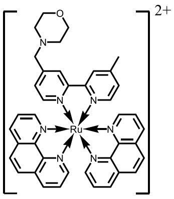	CH_3OH	450nm	0.631	12
40	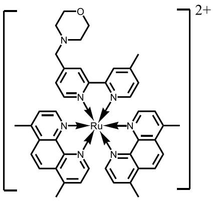	CH_3OH	450nm	0.438	12
41	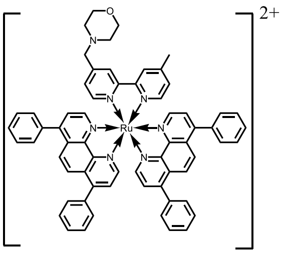	CH_3OH	450nm	0.866	12

42	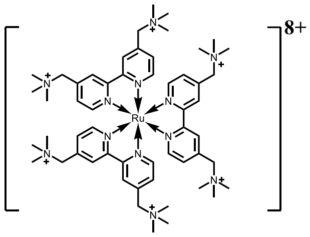	CH_3OH	450nm	0.99	13
43	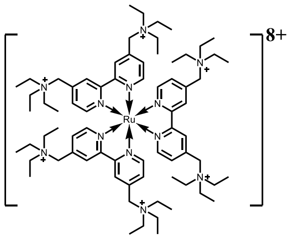	CH_3OH	450nm	0.95	13
44	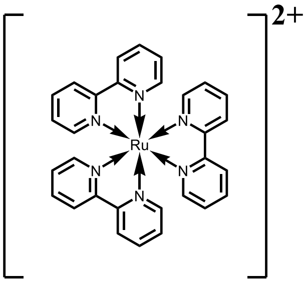	CH_3CN	450nm	0.54	14
45	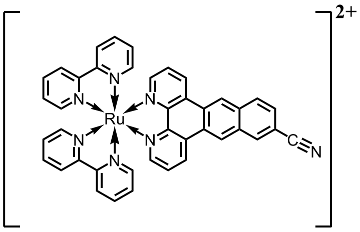	CH_3CN	420nm	0.207	15
		CH_3CN	575nm	0.111	15
46	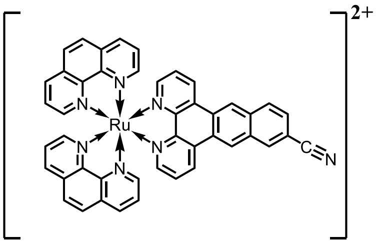	CH_3CN	420nm	0.201	15
		CH_3CN	575nm	0.140	15
47	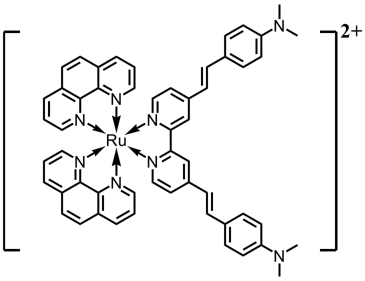	CH_3CN	450nm	0.62	16
		CH_3CN	540nm	0.57	16

48	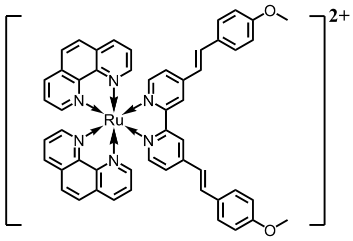	CH_3CN	450nm	0.92	16
		CH_3CN	540nm	0.83	16
49	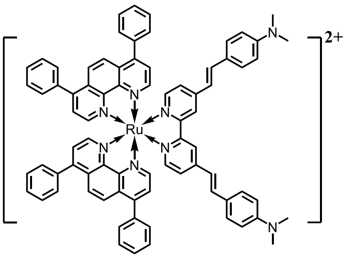	CH_3CN	450nm	0.48	16
		CH_3CN	540nm	0.43	16
50	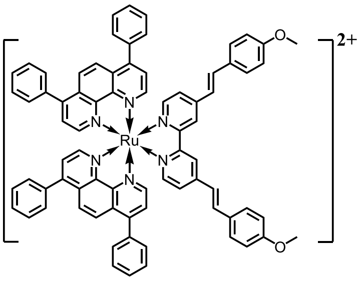	CH_3CN	450nm	0.76	16
		CH_3CN	540nm	0.77	16
51	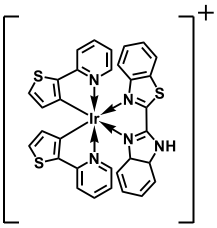	CH_3CN	405nm	0.40	17
52	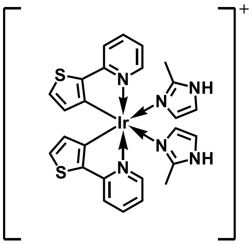	CH_3CN	405nm	0.82	17
53	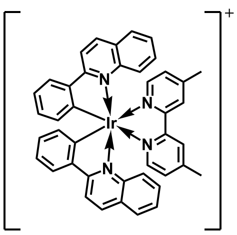	CH_3CN	365nm	0.47	18

54	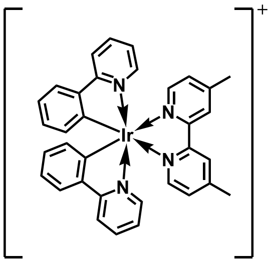	CH_3CN	365nm	0.53	18
55	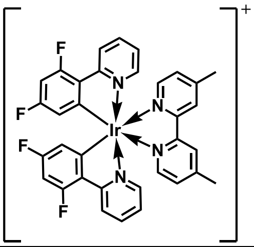	CH_3CN	365nm	0.47	18
56	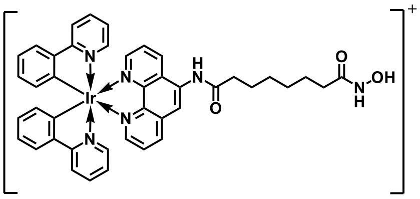	DMSO	365nm	0.45	19
		DMSO	425nm	0.24	19
57	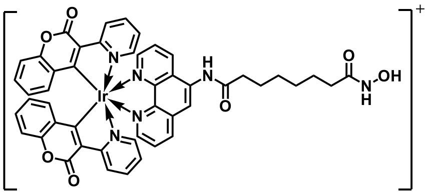	DMSO	365nm	0.38	19
		DMSO	425nm	0.17	19
58	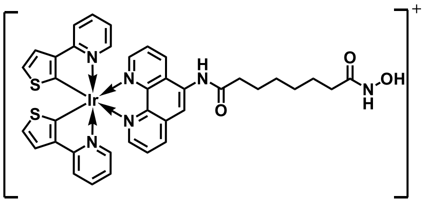	DMSO	365nm	0.21	19
		DMSO	425nm	0.14	19
59	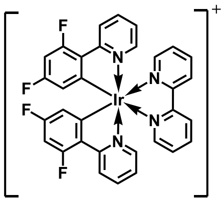	H_2O	354nm	0.32	20
60	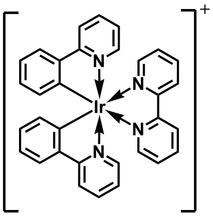	H_2O	374nm	0.37	20

61	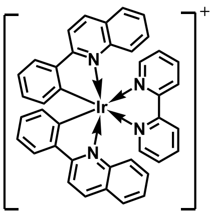	H_2O	432nm	0.95	20
62	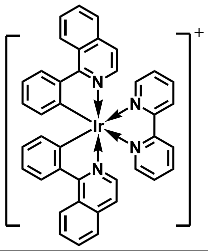	H_2O	437nm	0.78	20
63	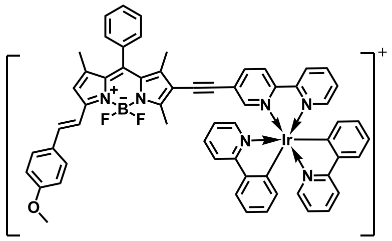	CH_2Cl_2	611nm	0.53	21
64	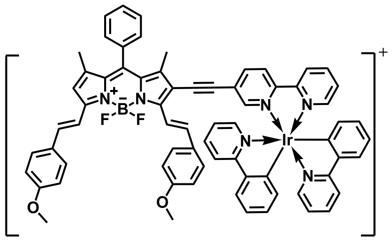	CH_2Cl_2	652nm	0.81	21
65	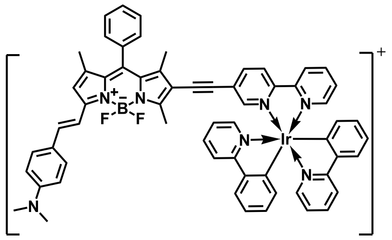	CH_2Cl_2	642nm	0.06	21
66	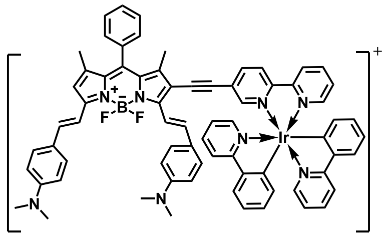	CH_2Cl_2	664nm	0.02	21

67	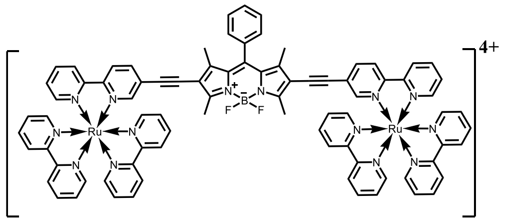	CH_2Cl_2	540nm	0.794	22
68	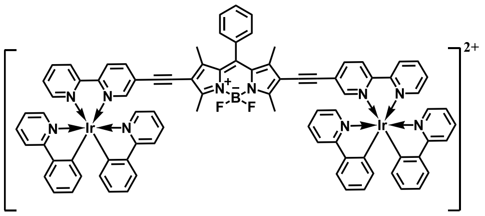	CH_2Cl_2	540nm	0.749	22
69	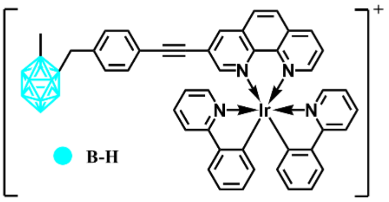	CH_3CN	355nm	0.25	5
70	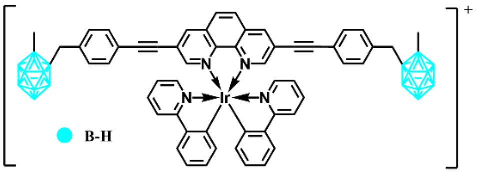	CH_3CN	355nm	0.10	5
71	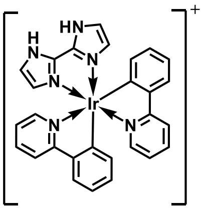	DMSO	405nm	0.17	23
72	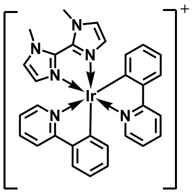	DMSO	405nm	0.28	23
73	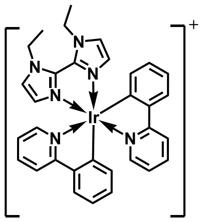	DMSO	405nm	0.21	23

74	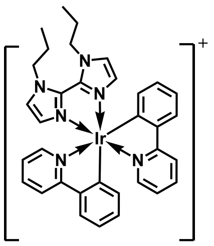	DMSO	405nm	0.51	[23]
75	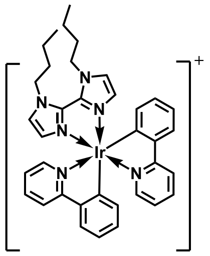	DMSO	405nm	0.59	[23]
76	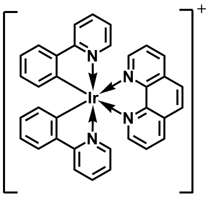	H_2O	800nm	0.036	[24]
		CH_2Cl_2	800nm	0.53	[24]
77	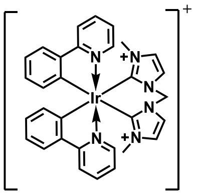	DMSO	365nm	0.58	[25]
78	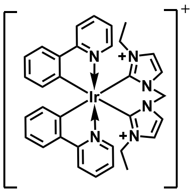	DMSO	365nm	0.59	[25]
79	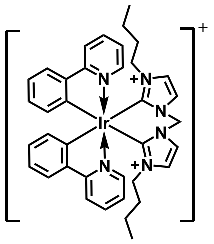	DMSO	365nm	0.62	[25]
80	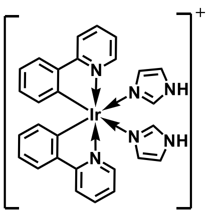	CH_3OH	425nm	0.80	[26]

81	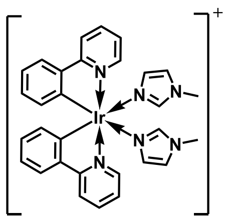	CH_3OH	425nm	0.79	26
82	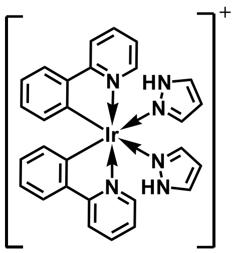	CH_3OH	425nm	0.86	26
83	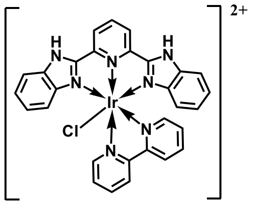	H_2O	425nm	0.023	27
84	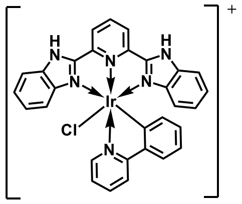	H_2O	425nm	0.072	27
85	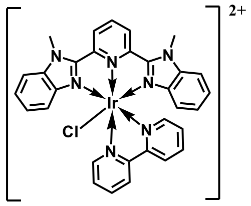	H_2O	425nm	0.330	27
86	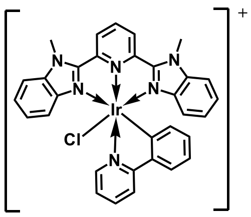	H_2O	425nm	0.170	27
87	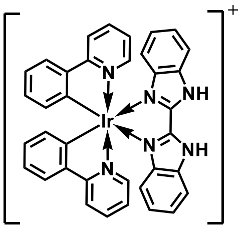	CH_2Cl_2	355nm	0.42	28

88	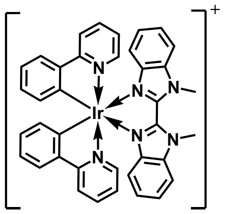	CH_2Cl_2	355nm	0.40	[28]
89	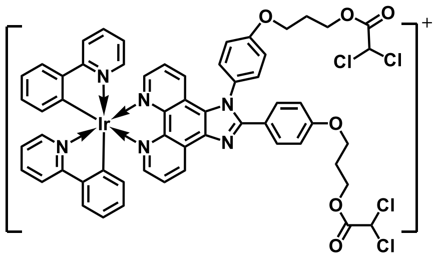	CH_3OH	405nm	0.68	[29]
90	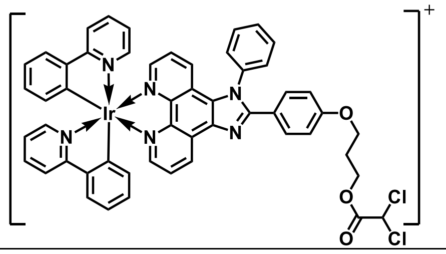	CH_3OH	405nm	0.74	[29]
91	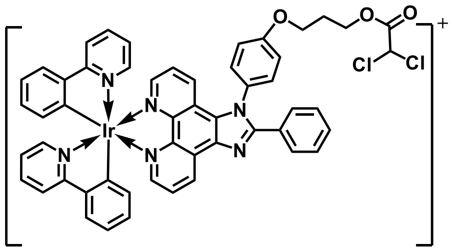	CH_3OH	405nm	0.70	[29]
92	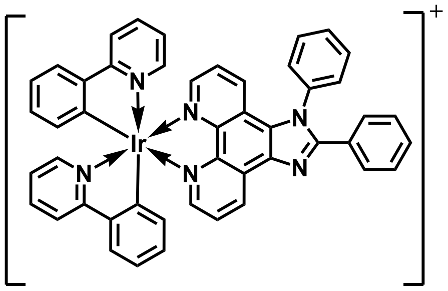	CH_3OH	405nm	0.53	[29]
93	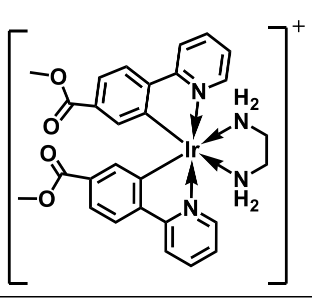	DMSO	365nm	0.29	[30]

94	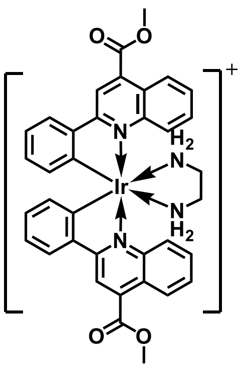	DMSO	365nm	0.082	30
95	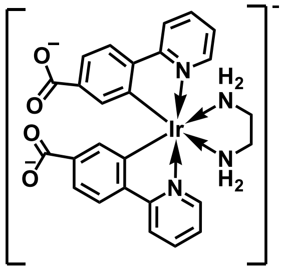	DMSO	365nm	0.27	30
96	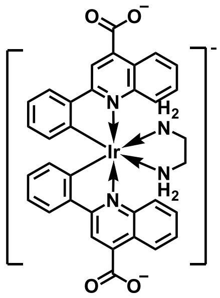	DMSO	365nm	0.21	30
97	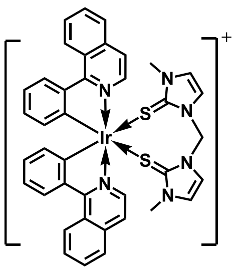	H_2O	465nm	0.73	31
98	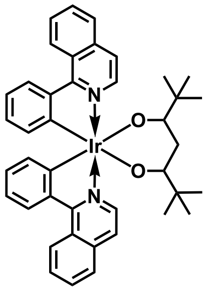	H_2O	465nm	0.81	31

99	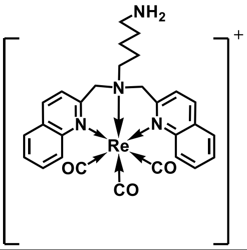	H_2O	350nm	0.26	32
		CH_3CN	350nm	0.75	32
100	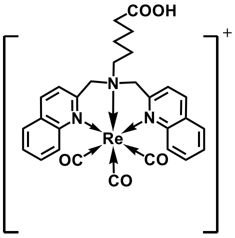	H_2O	350nm	0.24	32
		CH_3CN	350nm	0.72	32
101	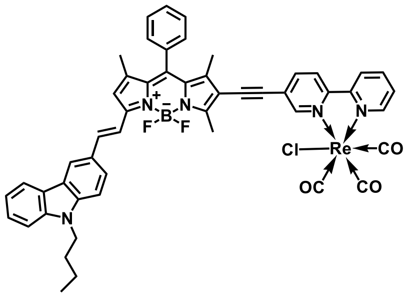	CH_2Cl_2	532nm	0.16	33
102	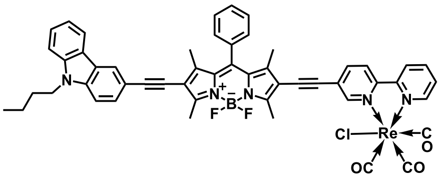	CH_2Cl_2	532nm	0.06	33
103	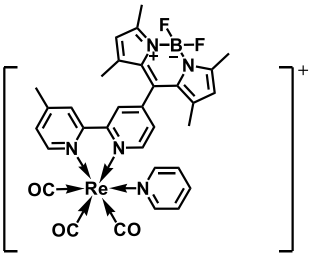	CH_3OH	525nm	0.89	34
104	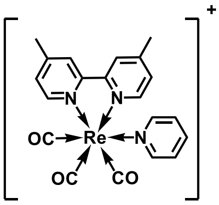	CH_3OH	425nm	0.59	34

Table S2. The detail of TMC photosensitizers data in external test set

Number	Structure	Solvent	Irradiation wavelength	Singlet oxygen quantum yield	Reference
1	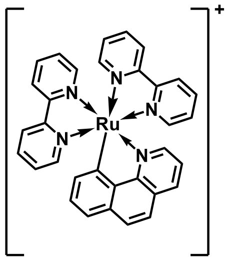	CH_3CN	543nm	0.068	35
2	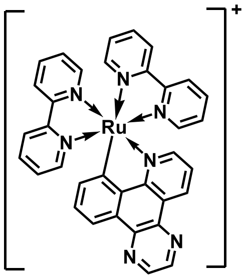	CH_3CN	532nm	0.076	35
3	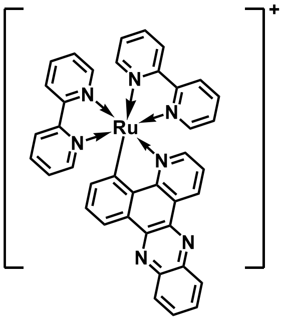	CH_3CN	528nm	0.013	35
4	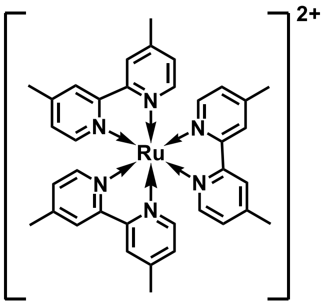	CH_3CN	450nm	0.66	14

5	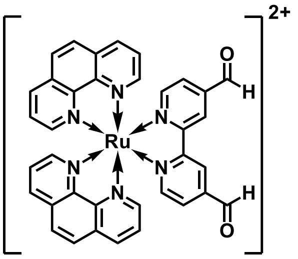	CH_3CN	450nm	0.64	36
6	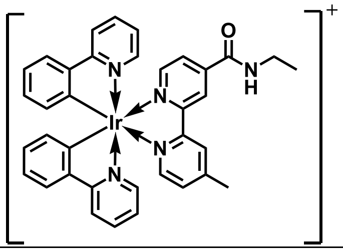	DMSO	365nm	0.38	37
7	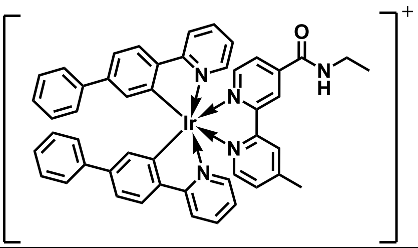	DMSO	365nm	0.50	37
8	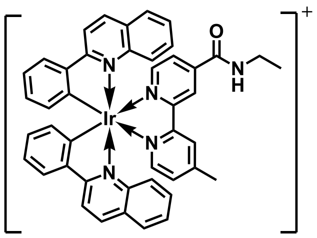	DMSO	365nm	0.58	37
9	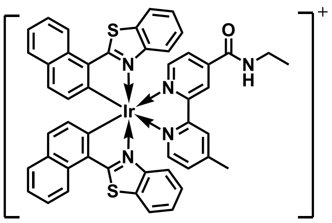	DMSO	365nm	0.83	37
10	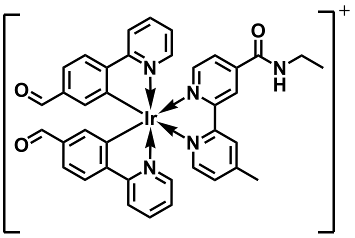	DMSO	365nm	0.79	37

11	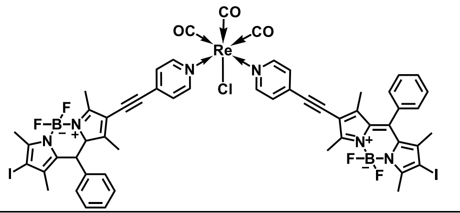	CH_3CN	532nm	0.77	38

Table S3. The performances of single-ML models with a kind of descriptors removed

Model	$R^2(Q^2)$	All descriptors	QCD removed	MSD removed	MCD removed	ECD removed
SVR	Training set	0.990	0.448	0.894	0.983	0.914
	Test set	0.935	0.775	0.699	0.899	0.947
	External test set	0.830	0.051	0.368	0.803	0.760
	LOO cross validation	0.579	0.398	0.540	0.571	0.531
KRR	Training set	0.993	0.896	0.934	0.989	0.952
	Test set	0.944	0.785	0.742	0.909	0.906
	External test set	0.747	0.214	0.160	0.686	0.653
	LOO cross validation	0.593	0.438	0.566	0.607	0.573
GPR	Training set	0.967	0.545	0.929	0.963	0.912
	Test set	0.907	0.664	0.729	0.870	0.867
	External test set	0.451	-0.142	0.135	0.461	0.462
	LOO cross validation	0.621	0.414	0.557	0.633	0.607
XGBoost	Training set	1.000	1.000	1.000	0.999	0.986
	Test set	0.729	0.890	0.682	0.788	0.659
	External test set	0.594	0.367	0.647	0.576	0.525
	LOO cross validation	0.587	0.608	0.629	0.560	0.539
RFR	Training set	0.900	0.917	0.908	0.889	0.888
	Test set	0.469	0.830	0.705	0.646	0.568
	External test set	0.765	0.363	0.546	0.718	0.618
	LOO cross validation	0.540	0.490	0.476	0.481	0.490
KNR	Training set	1.000	1.000	1.000	1.000	0.988
	Test set	0.834	0.749	0758	0.830	0.669
	External test set	0.483	0.408	0.283	0.508	0.514
	LOO cross validation	0.538	0.461	0.487	0.545	0.477

Table S4. the $R^2(Q^2)$ result of GPR, XGBoost, RFR and KNR in descriptors filter

Model	R2(Q2)	30 descriptors	35 descriptors	40 descriptors	45 descriptors	50 descriptors	All descriptors
GPR	Training set	0.988	0.988	0.989	0.987	0.984	0.967
	Test set	0.775	0.827	0.866	0.870	0.875	0.907
	External test set	0.507	0.636	0.701	0.665	0.663	0.451
	LOO cross validation	0.654	0.619	0.647	0.657	0.649	0.621
XGBoost	Training set	0.995	1.000	0.996	0.999	1.000	1.000
	Test set	0.829	0.861	0.818	0.884	0.790	0.729
	External test set	0.625	0.632	0.496	0.555	0.570	0.594
	LOO cross validation	0.592	0.590	0.639	0.635	0.576	0.587
RFR	Training set	0.941	0.931	0.939	0.926	0.933	0.900
	Test set	0.732	0.679	0.591	0.581	0.697	0.469
	External test set	0.342	0.428	0.403	0.490	0.454	0.765
	LOO cross validation	0.571	0.534	0.570	0.588	0.534	0.540
KNR	Training set	1.000	1.000	1.000	1.000	1.000	1.000
	Test set	0.853	0.825	0.782	0.819	0.809	0.834
	External test set	0.261	0.272	0.234	0.302	0.269	0.483
	LOO cross validation	0.513	0.493	0.517	0.471	0.490	0.538

Table S5. The delta-learning model comparison on specialized TMC photosensitizers

Predicted set	Model		R2(Q2)	MaxAE	MAE	MSE
Ru-complex	Trained on all TM-complex	Train set	0.994	0.130	0.011	0.0005
		Test set	0.996	0.057	0.012	0.0005
		External test set	0.814	0.206	0.092	0.016
		LOO cross validation	0.801	0.407	0.088	0.017
	Trained on Ru-complex	Train set	0.995	0.069	0.016	0.0005
		Test set	0.930	0.216	0.067	0.009
		External test set	0.884	0.162	0.091	0.010
		LOO cross validation	0.811	0.479	0.084	0.016
Ir-complex	Trained on all TM-complex	Train set	0.994	0.132	0.007	0.0004
		Test set	1.000	0.009	0.003	0.00002
		External test set	0.414	0.256	0.099	0.017
		LOO cross validation	0.331	0.651	0.154	0.046
	Trained on Ir-complex	Train set	1.000	0.008	0.001	0.000007
		Test set	0.955	0.110	0.040	0.003
		External test set	0.417	0.251	0.105	0.017
		LOO cross validation	0.382	0.584	0.145	0.042

Table S6. The Mixture-of-Experts model comparison on specialized TMC photosensitizers

Predicted set	Model		R2(Q2)	MaxAE	MAE	MSE
Ru-complex	Trained on all TM-complex	Train set	0.989	0.062	0.028	0.001
		Test set	0.979	0.100	0.043	0.002
		External test set	0.864	0.211	0.080	0.012
		LOO cross validation	0.730	0.585	0.101	0.023
	Trained on Ru-complex	Train set	0.997	0.042	0.011	0.0002
		Test set	0.937	0.182	0.072	0.008
		External test set	0.930	0.141	0.064	0.006
		LOO cross validation	0.715	0.543	0.094	0.025
Ir-complex	Trained on all TM-complex	Train set	0.979	0.132	0.028	0.001
		Test set	0.990	0.036	0.022	0.0006
		External test set	0.602	0.162	0.090	0.012
		LOO cross validation	0.340	0.500	0.157	0.045
	Trained on Ir-complex	Train set	0.986	0.090	0.025	0.001
		Test set	0.963	0.104	0.034	0.002
		External test set	0.609	0.219	0.079	0.011
		LOO cross validation	0.447	0.545	0.140	0.038

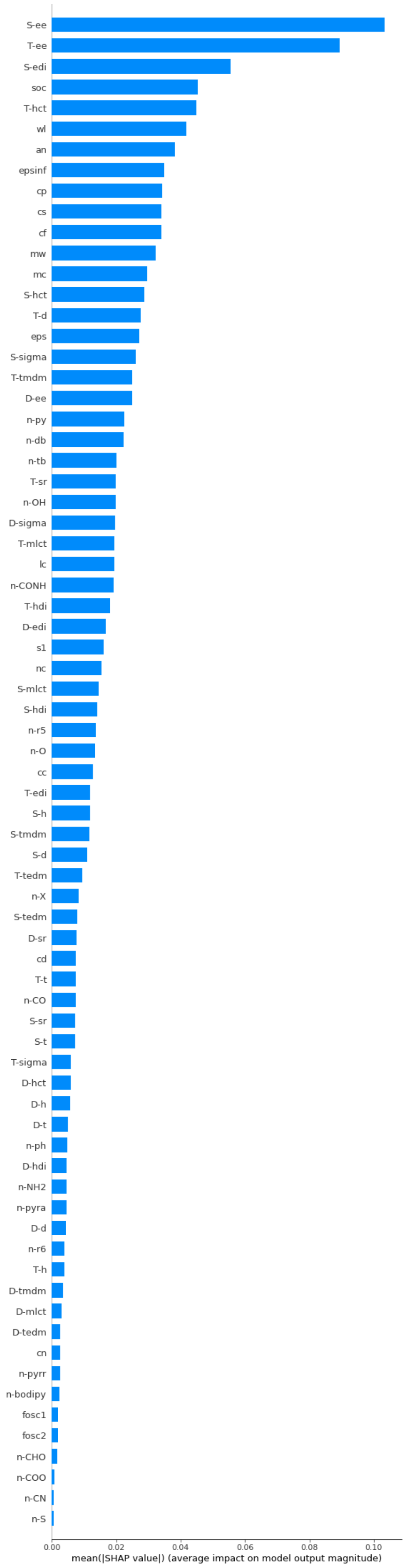

Figure S1. SHAP analysis of SVR model with all descriptors

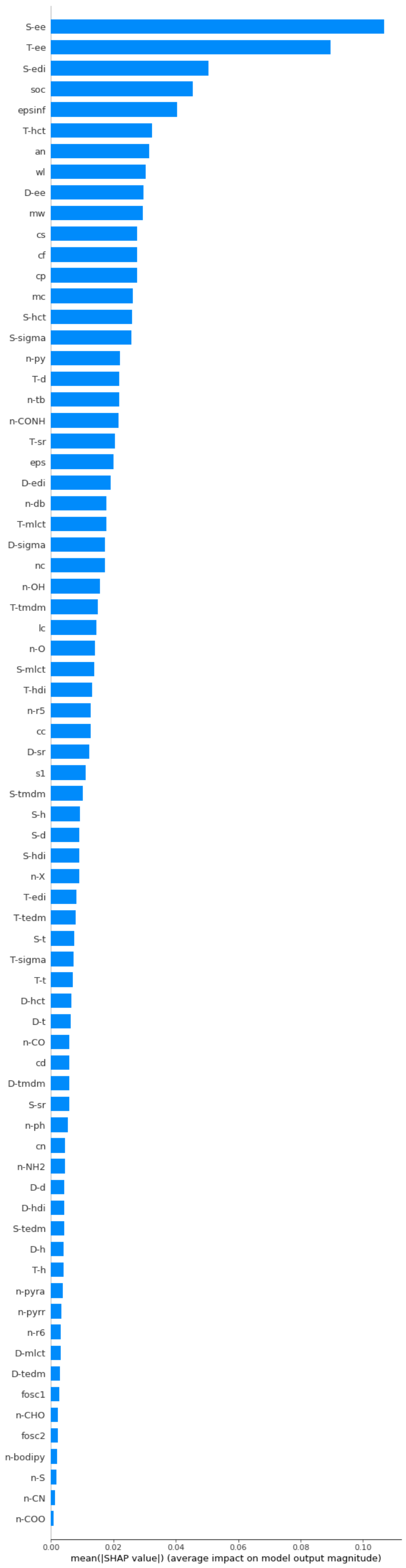

Figure S2. SHAP analysis of KRR model with all descriptors

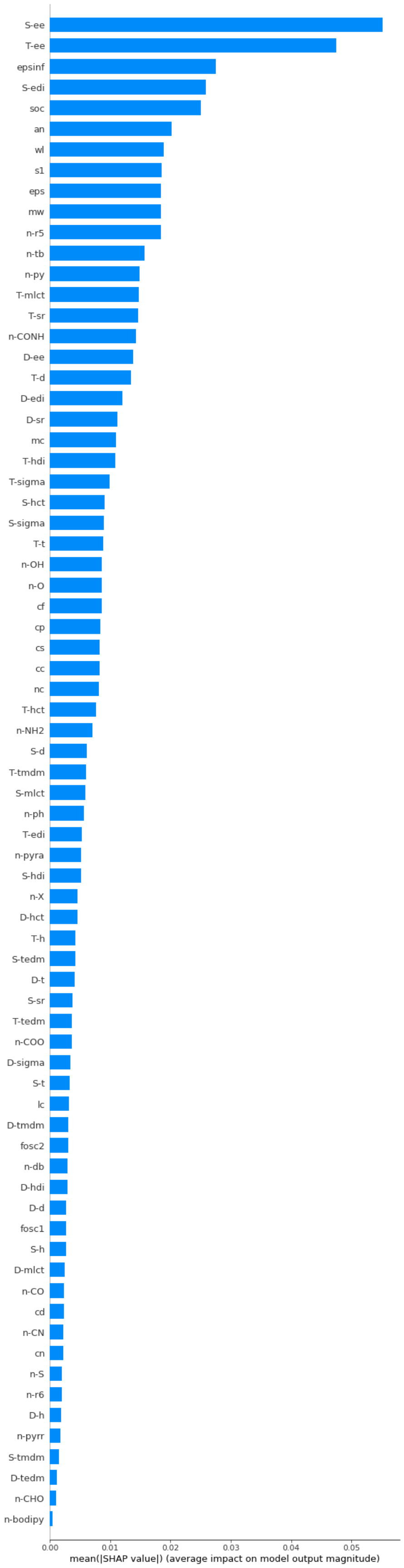

Figure S3. SHAP analysis of GPR model with all descriptors

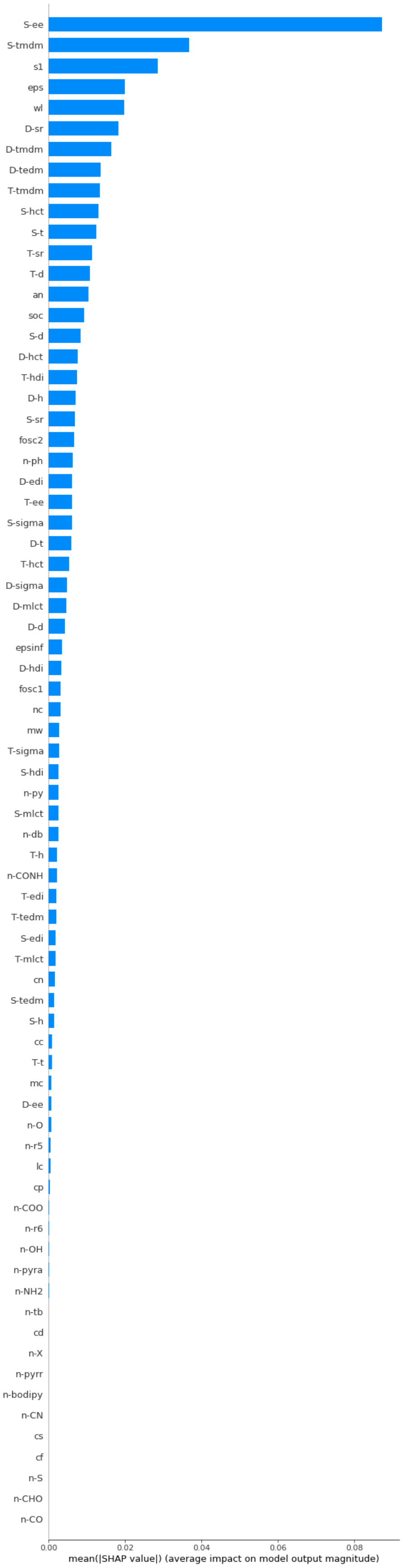

Figure S4. SHAP analysis of XGBoost model with all descriptors

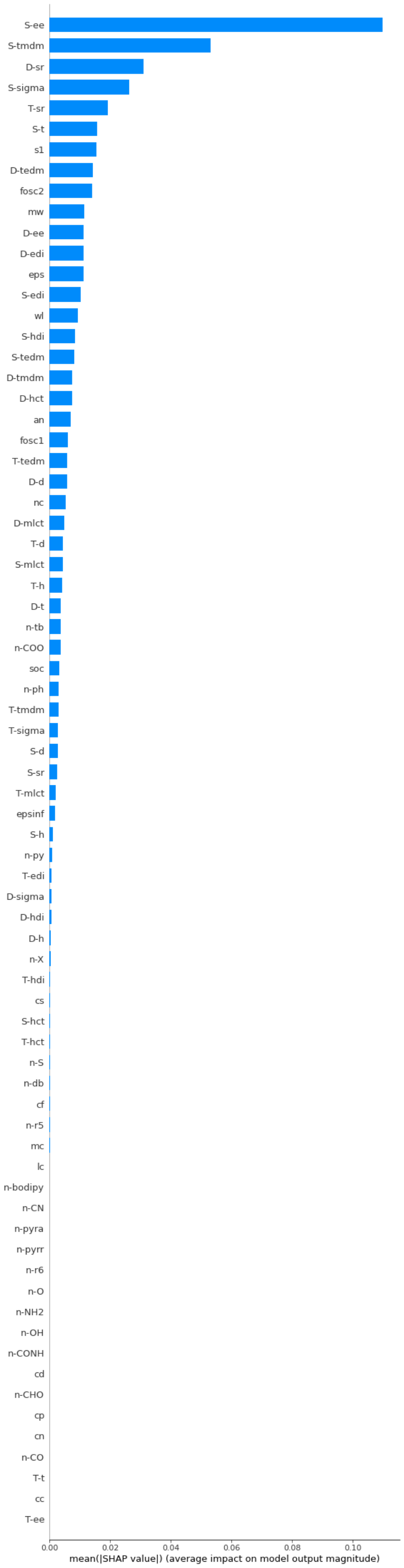

Figure S5. SHAP analysis of RFR model with all descriptors

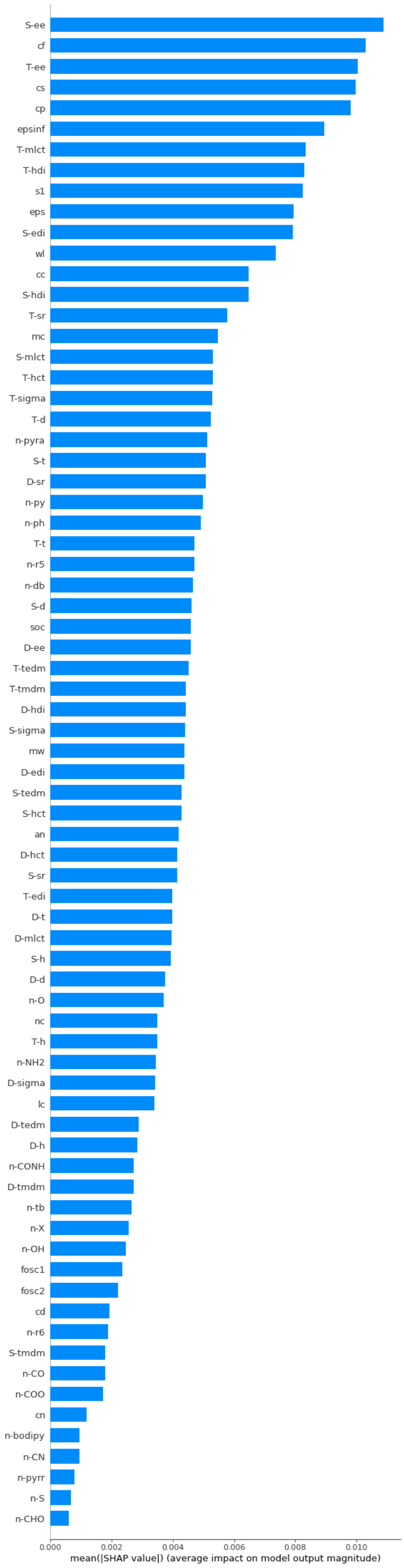

Figure S6. SHAP analysis of KNR model with all descriptors

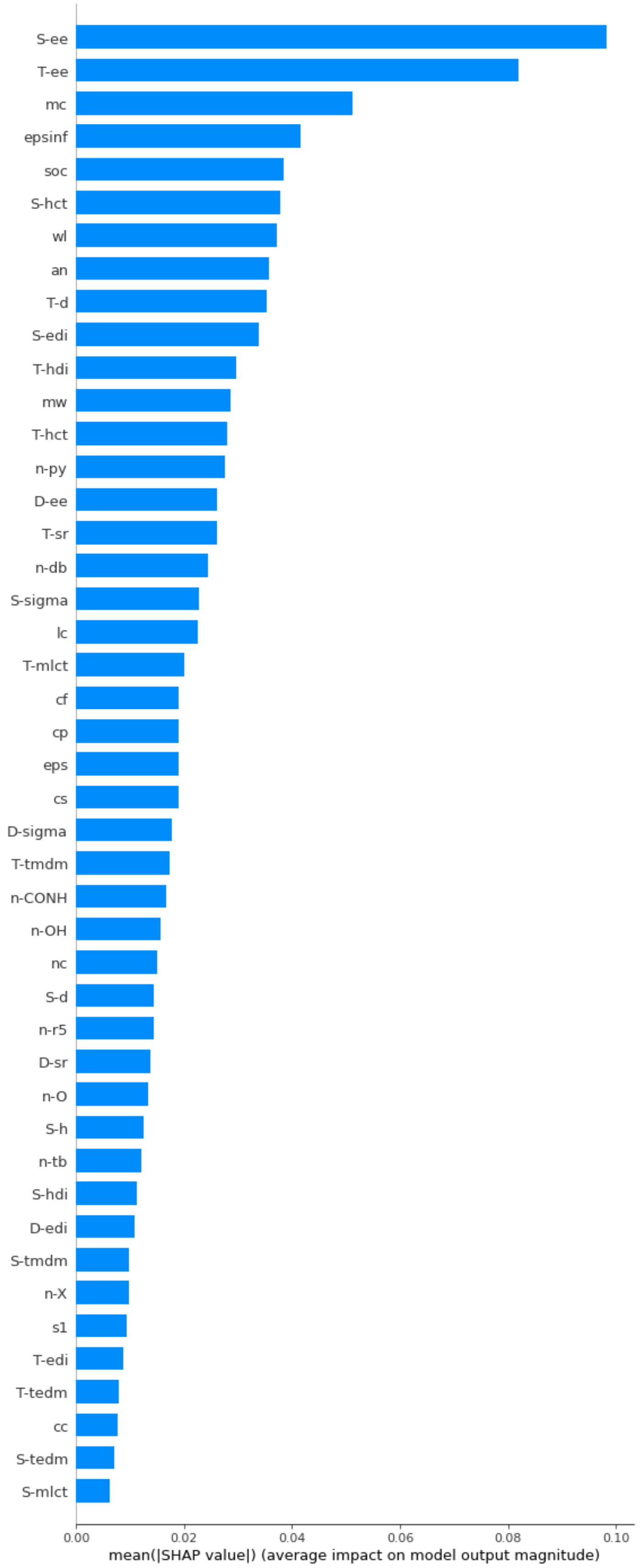

Figure S7. SHAP analysis of SVR model with filtered descriptors

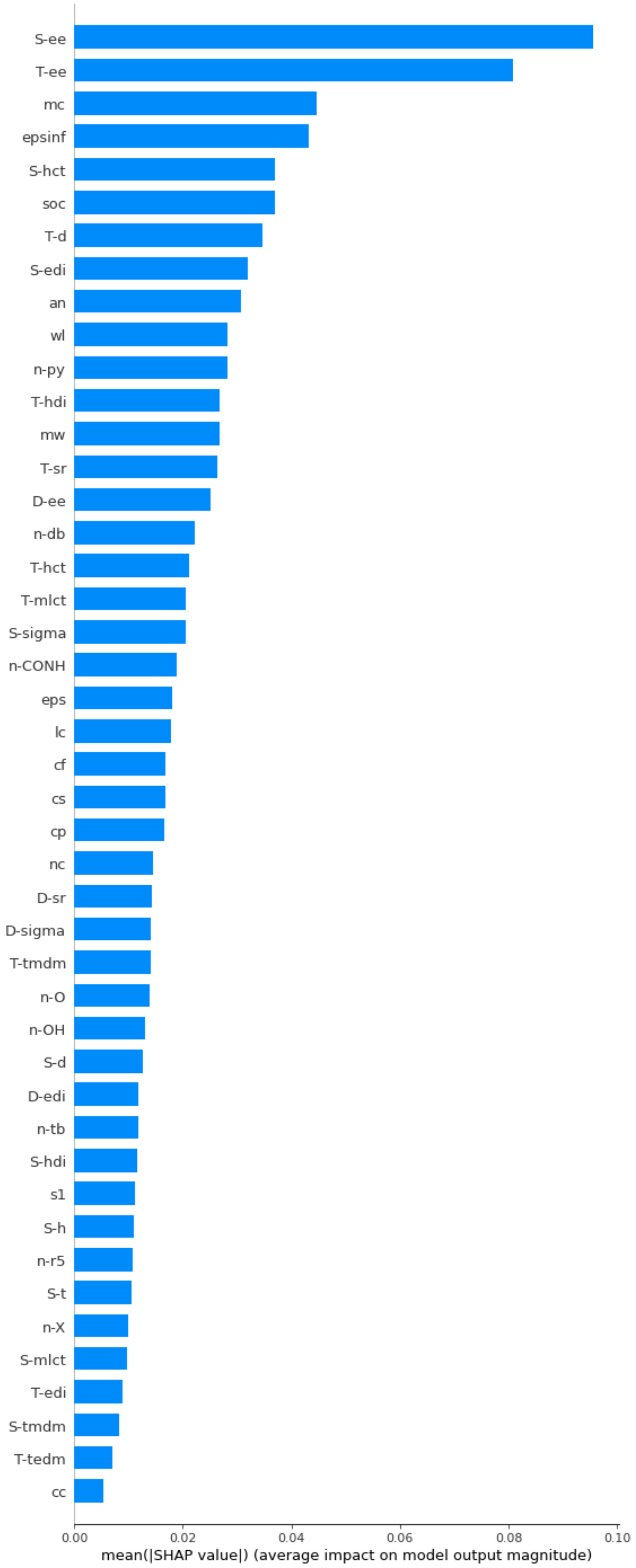

Figure S8. SHAP analysis of KRR model with filtered descriptors

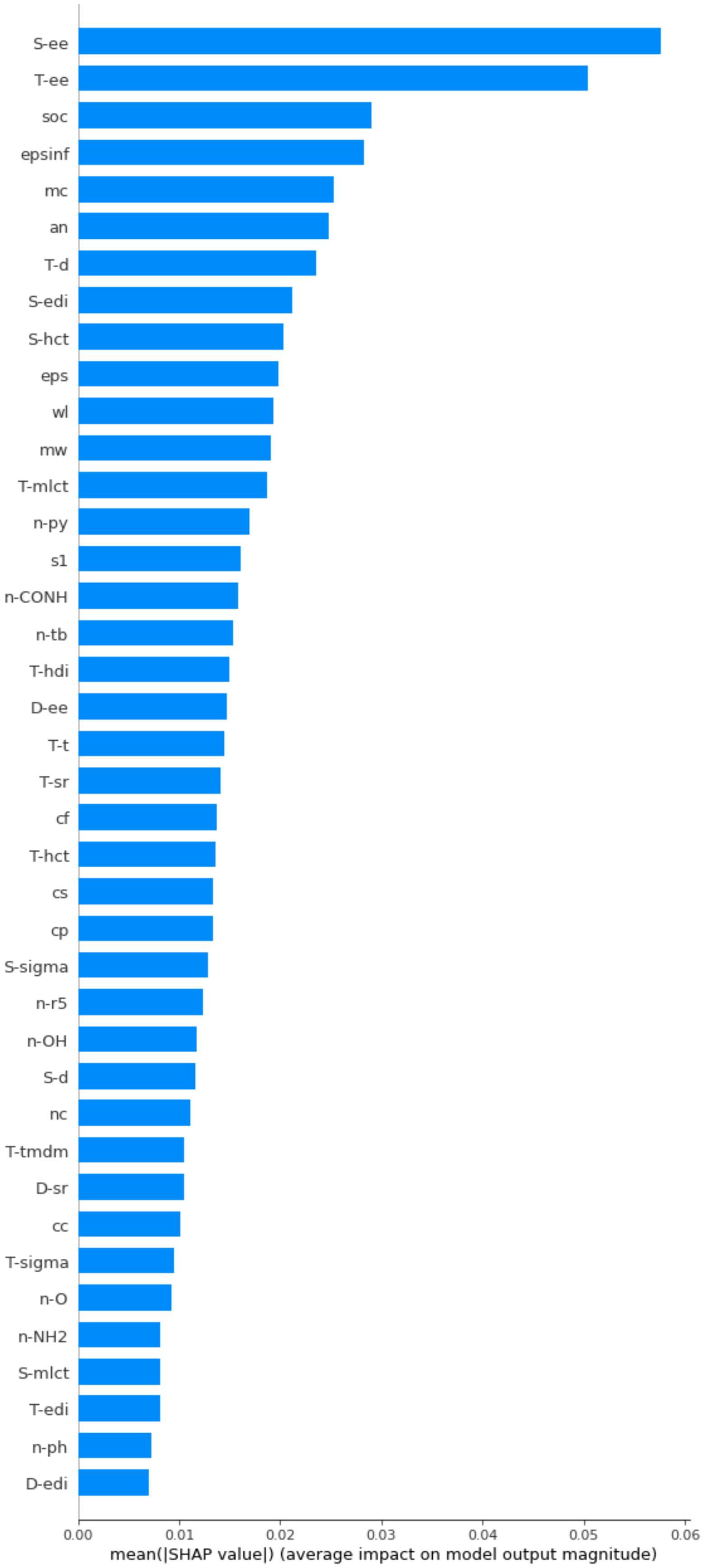

Figure S9. SHAP analysis of GPR model with filtered descriptors

The optimized hyperparameters of machine learning models

Random Seeds and Data Splitting: Training set accounts for 90% (122 data points) and test set accounts for 10% (14 data points) with random seed 430.
Cross-Validation Procedures: Leave-one-out cross validation (LOOCV) was used on the training set to test the stability of the model. For a dataset with N samples, LOOCV generates N unique folds. In each iteration, the test set consists of exactly one single sample, and the training set consists of the remaining N-1 samples. This process is repeated until every sample in the dataset has been used once as the test object.

Workflow: The workflow of single-ML model is added to SI as following:

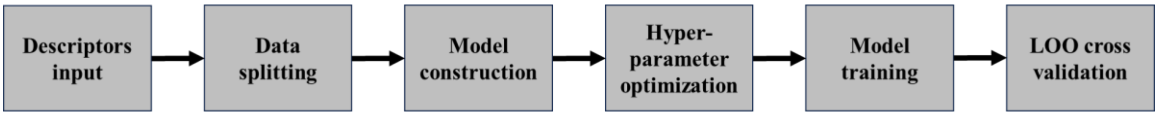

SVR model with best 45 descriptors: penalty coefficient- 29.558554549296286; tolerance- 0.024722705347541427; kernel type- ‘rbf’.
KRR model with best 45 descriptors: regularization parameter- 0.0019831474571510676; hyperparameter of gaussian kernel- 0.2492623291698518.
GPR model with best 40 descriptors: noise variance- 0.002480185388927965; kernel length scale- 1.3546909041856245; number of optimizers- 14.
Delta-learning model:

SVR model: penalty coefficient- 29.1565347237523; tolerance- 0.025415003429245398; kernel type- ‘rbf’.

KRR model: regularization parameter- 0.0988267565923517; hyperparameter of gaussian kernel- 14.353564023202035.

Mixture-of-SVR-KRR-GPR model:

SVR model: penalty coefficient- 0.001224920796736083; tolerance- 0.5106708941365737; kernel type- ‘rbf’.

KRR model: regularization parameter- 1.0060102766515068e-06; hyperparameter of gaussian kernel- 0.004584693453481658.

GPR model: noise variance- 0.0010754201112731737; kernel length scale- 26.362584365911015; number of optimizers- 6.

Weight of SVR model- 0.06972438202684254; weight of KRR model- 0.43549802468003523; weight of GPR model- 0.49477759329312226.

Mixture-of-SVR-KRR model:

SVR model: penalty coefficient- 26.3550844585928; tolerance- 0.029066406249881244; kernel type- ‘rbf’.

KRR model: regularization parameter- 1.005467085515253e-06; hyperparameter of

gaussian kernel- 0.004254382989116614.
Weight of SVR model- 0.7822341627717817; weight of KRR model- 0.2177658372282183.

REFERENCES

(1) Zhang, Y.; Zhou, Q.; Tian, N.; Li, C.; Wang, X. Ru (II)-complex-based DNA photocleaver having intense absorption in the phototherapeutic window. *Inorg. Chem.* **2017**, *56*(4), 1865-1873, DOI: 10.1021/acs.inorgchem.6b02459.

(2) Li, S.; Zhao, J.; Wang, X.; Xu, G.; Gou, S.; Zhao, Q. Design of a tris-heteroleptic Ru (II) complex with red-light excitation and remarkably improved photobiological activity. *Inorg. Chem.* **2020**, *59*(15), 11193-11204, DOI: 10.1021/acs.inorgchem.0c01860.

(3) Ortega-Forte, E.; Rovira, A.; Lopez-Corrales, M.; Hernandez-Garcia, A.; Ballester, F. J.; Izquierdo-Garcia, E.; Jorda-Redondo, M.; Bosch, M.; Nonell, S.; Santana, M. D.; et al. A near-infrared light-activatable ru(ii)-coumarin photosensitizer active under hypoxic conditions. *Chem. Sci.* **2023**, *14*(26), 7170-7184, DOI: 10.1039/d3sc01844j.

(4) Mazuryk, O.; Janczy-Cempa, E.; Lagosz, J.; Rutkowska-Zbik, D.; Machnicka, A.; Krasowska, A.; Pietrzyk, P.; Stochel, G.; Brindell, M. Relevance of the electron transfer pathway in photodynamic activity of ru(II) polypyridyl complexes containing 4,7-diphenyl-1,10-phenanthroline ligands under normoxic and hypoxic conditions. *Dalton Trans.* **2022**, *51*(5), 1888-1900, DOI: 10.1039/d1dt02908h.

(5) Conway-Kenny, R.; Ferrer-Ugalde, A.; Careta, O.; Cui, X.; Zhao, J.; Nogues, C.; Nunez, R.; Cabrera-Gonzalez, J.; Draper, S. M. Ru(ii) and ir(iii) phenanthroline-based photosensitisers bearing o-carborane: PDT agents with boron carriers for potential BNCT. *Biomater. Sci.* **2021**, *9*(16), 5691-5702, DOI: 10.1039/d1bm00730k.

(6) Yang, J.; Cao, Q.; Hu, W. L.; Ye, R. R.; He, L.; Ji, L. N.; Qin, P. Z.; Mao, Z. W. Theranostic TEMPO-functionalized ru(ii) complexes as photosensitizers and oxidative stress indicators. *Dalton Trans.* **2017**, *46*(2), 445-454, DOI: 10.1039/c6dt04028d.

(7) Lv, Z.; Wei, H.; Li, Q.; Su, X.; Liu, S.; Zhang, K. Y.; Lv, W.; Zhao, Q.; Li, X.; Huang, W. Achieving efficient photodynamic therapy under both normoxia and hypoxia using cyclometalated ru(ii) photosensitizer through type i photochemical process. *Chem. Sci.* **2018**, *9*(2), 502-512, DOI: 10.1039/c7sc03765a.

(8) Butera, V.; Mazzone, G.; Detz, H. Dinuclear ruthenium (II)-pyrrolide complexes linked by different organic units as PDT photosensitizers: computational study of the linker influence on the photophysical properties*. *ChemPhotoChem* **2022**, *6*(10), DOI: 10.1002/cptc.202200094.

(9) Liu, X.; Li, G.; Xie, M.; Guo, S.; Zhao, W.; Li, F.; Liu, S.; Zhao, Q. Rational design of type i photosensitizers based on ru(ii) complexes for effective photodynamic therapy under hypoxia. *Dalton Trans.* **2020**, *49*(32), 11192-11200, DOI: 10.1039/d0dt01684e.

(10) Lifshits, L. M.; Roque, I. J.; Konda, P.; Monro, S.; Cole, H. D.; von Dohlen, D.; Kim, S.; Deep, G.; Thummel, R. P.; Cameron, C. G.; et al. Near-infrared absorbing ru(ii) complexes act as immunoprotective photodynamic therapy (PDT) agents against aggressive melanoma. *Chem. Sci.* **2020**, *11*(43), 11740-11762, DOI: 10.1039/d0sc03875j.

(11) Lincoln, R.; Kohler, L.; Monro, S.; Yin, H.; Stephenson, M.; Zong, R.; Chouai, A.; Dorsey, C.; Hennigar, R.; Thummel, R. P.; et al. Exploitation of long-lived 3IL excited states for metal–organic photodynamic therapy: verification in a metastatic melanoma model. *J. Am. Chem. Soc.* **2013**, *135*(45), 17161-17175, DOI: 10.1021/ja408426z.

(12) Qiu, K.; Wen, Y.; Ouyang, C.; Liao, X.; Liu, C.; Rees, T. W.; Zhang, Q.; Ji, L.; Chao, H. The stepwise photodamage of organelles by two-photon luminescent ruthenium(ii) photosensitizers. *Chem. Commun.* **2019**, *55*(75), 11235-11238, DOI: 10.1039/c9cc05962h.
(13) Huang, H.; Yu, B.; Zhang, P.; Huang, J.; Chen, Y.; Gasser, G.; Ji, L.; Chao, H. Highly charged ruthenium(II) polypyridyl complexes as lysosome-localized photosensitizers for two-photon photodynamic therapy. *Angew. Chem.-Int. Edit.* **2015**, *54*(47), 14049-14052, DOI: 10.1002/anie.201507800.
(14) Karges, J.; Blacque, O.; Goldner, P.; Chao, H.; Gasser, G. Towards long wavelength absorbing photodynamic therapy photosensitizers via the extension of a [ru(bipy)3]2+ core. *Eur. J. Inorg. Chem.* **2019**, *2019*(32), 3704-3712, DOI: 10.1002/ejic.201900569.
(15) Mari, C.; Rubbiani, R.; Gasser, G. Biological evaluation of nitrile containing ru(II) polypyridyl complexes as potential photodynamic therapy agents. *Inorganica Chim. Acta* **2017**, *454*, 21-26, DOI: 10.1016/j.ica.2015.10.010.
(16) Karges, J.; Kuang, S.; Ong, Y. C.; Chao, H.; Gasser, G. One- and two-photon phototherapeutic effects of Ru^{II} polypyridine complexes in the hypoxic centre of large multicellular tumor spheroids and tumor-bearing mice**. *Chem.-Eur. J.* **2021**, *27*(1), 362-370, DOI: 10.1002/chem.202003486.
(17) Martínez-Alonso, M.; Jones, C. G.; Shipp, J. D.; Chekulaev, D.; Bryant, H. E.; Weinstein, J. A. Phototoxicity of cyclometallated ir(III) complexes bearing a thio-bis-benzimidazole ligand, and its monodentate analogue, as potential PDT photosensitisers in cancer cell killing. *JBIC Journal of Biological Inorganic Chemistry* **2024**, *29*(1), 113-125, DOI: 10.1007/s00775-023-02031-z.
(18) Estevão, B. M.; Vilela, R. R. C.; Geremias, I. P.; Zanoni, K. P. S.; de Camargo, A. S. S.; Zucolotto, V. Mesoporous silica nanoparticles incorporated with ir(III) complexes: from photophysics to photodynamic therapy. *Photodiagnosis Photodyn. Ther.* **2022**, *40*, 103052, DOI: 10.1016/j.pdpdt.2022.103052.
(19) Ye, R.; Tan, C.; He, L.; Chen, M.; Ji, L.; Mao, Z. Cyclometalated ir(iii) complexes as targeted theranostic anticancer therapeutics: combining HDAC inhibition with photodynamic therapy. *Chem. Commun.* **2014**, *50*(75), 10945, DOI: 10.1039/C4CC05215C.
(20) Nam, J. S.; Kang, M.; Kang, J.; Park, S.; Lee, S. J. C.; Kim, H.; Seo, J. K.; Kwon, O.; Lim, M. H.; Rhee, H.; et al. Endoplasmic reticulum-localized iridium(III) complexes as efficient photodynamic therapy agents via protein modifications. *J. Am. Chem. Soc.* **2016**, *138*(34), 10968-10977, DOI: 10.1021/jacs.6b05302.
(21) Majumdar, P.; Yuan, X.; Li, S.; Le Guennic, B.; Ma, J.; Zhang, C.; Jacquemin, D.; Zhao, J. Cyclometalated ir(iii) complexes with styryl-BODIPY ligands showing near IR absorption/emission: preparation, study of photophysical properties and application as photodynamic/luminescence imaging materials. *J. Mat. Chem. B* **2014**, *2*(19), 2838-2854, DOI: 10.1039/C4TB00284A.
(22) Wang, J.; Lu, Y.; Mcgoldrick, N.; Zhang, C.; Yang, W.; Zhao, J.; Draper, S. M. Dual phosphorescent dinuclear transition metal complexes, and their application as triplet photosensitizers for TTA upconversion and photodynamic therapy. *J. Mater. Chem. C* **2016**, *4*(25), 6131-6139, DOI: 10.1039/C6TC01926A.
(23) Ouyang, M.; Zeng, L.; Qiu, K.; Chen, Y.; Ji, L.; Chao, H. Cyclometalated Ir^{III} complexes as mitochondria-targeted photodynamic anticancer agents. *Eur. J. Inorg. Chem.* **2017**, *2017*(12), 1764-1771, DOI: 10.1002/ejic.201601129.
(24) Takizawa, S.; Breitenbach, T.; Westberg, M.; Holmegaard, L.; Gollmer, A.; Jensen, R. L.;

Murata, S.; Ogilby, P. R. Solvent dependent photosensitized singlet oxygen production from an ir(III) complex: pointing to problems in studies of singlet-oxygen-mediated cell death. *Photochem. Photobiol. Sci.* **2015**, *14*(10), 1831-1843, DOI: 10.1039/c5pp00230c.

(25) Li, Y.; Tan, C.; Zhang, W.; He, L.; Ji, L.; Mao, Z. Phosphorescent iridium(III)-bis-n-heterocyclic carbene complexes as mitochondria-targeted theranostic and photodynamic anticancer agents. *Biomaterials* **2015**, *39*, 95-104, DOI: 10.1016/j.biomaterials.2014.10.070.

(26) Wu, N.; Cao, J.; Wu, X.; Tan, C.; Ji, L.; Mao, Z. Iridium(iii) complexes with five-membered heterocyclic ligands for combined photodynamic therapy and photoactivated chemotherapy. *Dalton Trans.* **2017**, *46*(39), 13482-13491, DOI: 10.1039/c7dt02477k.

(27) Zheng, Y.; He, L.; Zhang, D.; Tan, C.; Ji, L.; Mao, Z. Mixed-ligand iridium(iii) complexes as photodynamic anticancer agents. *Dalton Trans.* **2017**, *46*(34), 11395-11407, DOI: 10.1039/c7dt02273e.

(28) Mckenzie, L. K.; Sazanovich, I. V.; Baggaley, E.; Bonneau, M.; Guerchais, V.; Williams, J. A. G.; Weinstein, J. A.; Bryant, H. E. Metal complexes for two-photon photodynamic therapy: a cyclometallated iridium complex induces two-photon photosensitization of cancer cells under near-IR light. *Chem.-Eur. J.* **2017**, *23*(2), 234-238, DOI: 10.1002/chem.201604792.

(29) Liu, J.; Jin, C.; Yuan, B.; Chen, Y.; Liu, X.; Ji, L.; Chao, H. Enhanced cancer therapy by the marriage of metabolic alteration and mitochondrial-targeted photodynamic therapy using cyclometalated Ir (III) complexes. *Chem. Commun.* **2017**, *53*(71), 9878-9881, DOI: 10.1039/C7CC05518H.

(30) Tang, T. S.; Leung, K.; Louie, M.; Liu, H.; Cheng, S. H.; Lo, K. K. Phosphorescent biscyclometallated iridium(III) ethylenediamine complexes functionalised with polar ester or carboxylate groups as bioimaging and visualisation reagents. *Dalton Trans.* **2015**, *44*(11), 4945-4956, DOI: 10.1039/C4DT02890B.

(31) Zhang, P.; Chiu, C. K. C.; Huang, H.; Lam, Y. P. Y.; Habtemariam, A.; Malcomson, T.; Paterson, M. J.; Clarkson, G. J.; O'Connor, P. B.; Chao, H.; et al. Organoiridium photosensitizers induce specific oxidative attack on proteins within cancer cells. *Angewandte Chemie* **2017**, *129*(47), 15094-15098, DOI: 10.1002/ange.201709082.

(32) Leonidova, A.; Pierroz, V.; Rubbiani, R.; Heier, J.; Ferrari, S.; Gasser, G. Towards cancer cell-specific phototoxic organometallic rhenium(I) complexes. *Dalton Trans.* **2014**, *43*(11), 4287-4294, DOI: 10.1039/C3DT51817E.

(33) Zhong, F.; Yuan, X.; Zhao, J.; Wang, Q. Visible light-harvesting tricarbonyl Re(I) complex: synthesis and application in intracellular photodynamic effect and luminescence imaging. *Sci. China Chem.* **2016**, *59*(1), 70-77, DOI: 10.1007/s11426-015-5491-x.

(34) Feng, W.; Liang, B.; Chen, B.; Liu, Q.; Pan, Z.; Liu, Y.; He, L. A tricarbonyl rhenium(I) complex decorated with boron dipyrromethene for endoplasmic reticulum-targeted photodynamic therapy. *Dyes Pigment.* **2023**, *211*, 111077, DOI: 10.1016/j.dyepig.2023.111077.

(35) Sainuddin, T.; Mccain, J.; Pinto, M.; Yin, H.; Gibson, J.; Hetu, M.; Mcfarland, S. A. Organometallic Ru (II) photosensitizers derived from π-expansive cyclometalating ligands: surprising theranostic PDT effects. *Inorg. Chem.* **2016**, *55*(1), 83-95, DOI: 10.1021/acs.inorgchem.5b01838.

(36) Karges, J.; Heinemann, F.; Maschietto, F.; Patra, M.; Blacque, O.; Ciofini, I.; Spingler, B.; Gasser, G. A ru(II) polypyridyl complex bearing aldehyde functions as a versatile synthetic precursor for long-wavelength absorbing photodynamic therapy photosensitizers. *Bioorg. Med. Chem.* **2019**, *27*(12), 2666-2675, DOI: 10.1016/j.bmc.2019.05.011.

(37) Li, S. P.; Lau, C. T.; Louie, M.; Lam, Y.; Cheng, S. H.; Lo, K. K. Mitochondria-targeting cyclometalated iridium(III)–PEG complexes with tunable photodynamic activity. *Biomaterials* **2013**, *34*(30), 7519-7532, DOI: 10.1016/j.biomaterials.2013.06.028.
(38) Tyagi, N.; Kaur, N.; Sahoo, S. C.; Venugopalan, P. Photodynamic therapy applications of re(i)-BODIPY functionalized nanoparticles. *Appl. Organomet. Chem.* **2022**, *36*(2), e6494, DOI: 10.1002/aoc.6494.
